# Halogen Bond‐Tuning of Responsive Supramolecular Amino Acid Hydrogels

**DOI:** 10.1002/chem.202501476

**Published:** 2025-08-17

**Authors:** Eleonora Veronese, Andrea Pizzi, Nicola Demitri, Greta Bergamaschi, Giancarlo Terraneo, Pierangelo Metrangolo, Valentina Dichiarante

**Affiliations:** ^1^ Laboratory of Supramolecular and Bio‐Nanomaterials (SBNLab) Department of Chemistry Materials and Chemical Engineering “Giulio Natta” Politecnico di Milano Via L. Mancinelli 7 Milan 20131 Italy; ^2^ Elettra Sincrotrone Trieste S.S. 14, km 163.5, in Area Science Park Basovizza‐Trieste 34149 Italy; ^3^ Istituto di Scienze e Tecnologie Chimiche National Research Council of Italy (SCITEC‐CNR) Via M. Bianco 9 Milan 20131 Italy

**Keywords:** fluorine, halogen bond, iodine, self‐assembly, supramolecular hydrogels

## Abstract

*N*‐Fmoc‐pentafluoro‐*L*‐phenylalanine (**F_5_**) forms stable supramolecular hydrogels, mainly through π‐π stacking of its aromatic groups. Hydrogen bonding‐driven co‐assembly of **F_5_** with suitable partner molecules has been reported to affect the gel's properties, in accordance with what is observed in their corresponding co‐crystal structures. Herein, we extended this hydrogel modulation strategy to halogen bonding (XB) interactions by introducing an XB‐donating iodine atom in the *para*‐position of the **F_5_** phenyl ring. Under conditions mimicking biological environments, both crystal packing and hydrogelation of the resulting *N*‐Fmoc‐4‐iodo‐tetrafluoro‐phenylalanine (**IF_4_**), were significantly affected by iodine‐π interactions. Slower fibril formation kinetics and reduced strength of **IF_4_** hydrogels in phosphate buffer solution, compared to **F_5_**, mirrored iodine‐induced changes in Fmoc stacking in the solid state. Notably, the addition of strong XB‐acceptors – such as iodide anions or pyridine‐containing substrates, like vitamin B_3_ – induced a significant increase in gel stiffness. These findings suggest the possibility of exploiting properly tailored Fmoc‐amino acids as “XB‐responsive” hydrogelators, useful for anion sensing applications or for trapping bioactive molecules.

## Graphical abstract

XB strongly influences both solid‐state packing and rheological properties of hydrogels of a tailored halogenated Fmoc‐amino acid in biomimetic environments. Interestingly, the addition of effective XB‐acceptors (Nu) to the gelation medium increased gel stiffness, preluding to the possible future development of XB‐responsive amino acid hydrogels in anion sensing, or for the encapsulation of specific guest molecules.

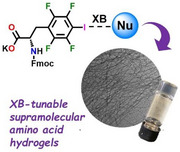

## 1 Introduction

**Figure 1:**
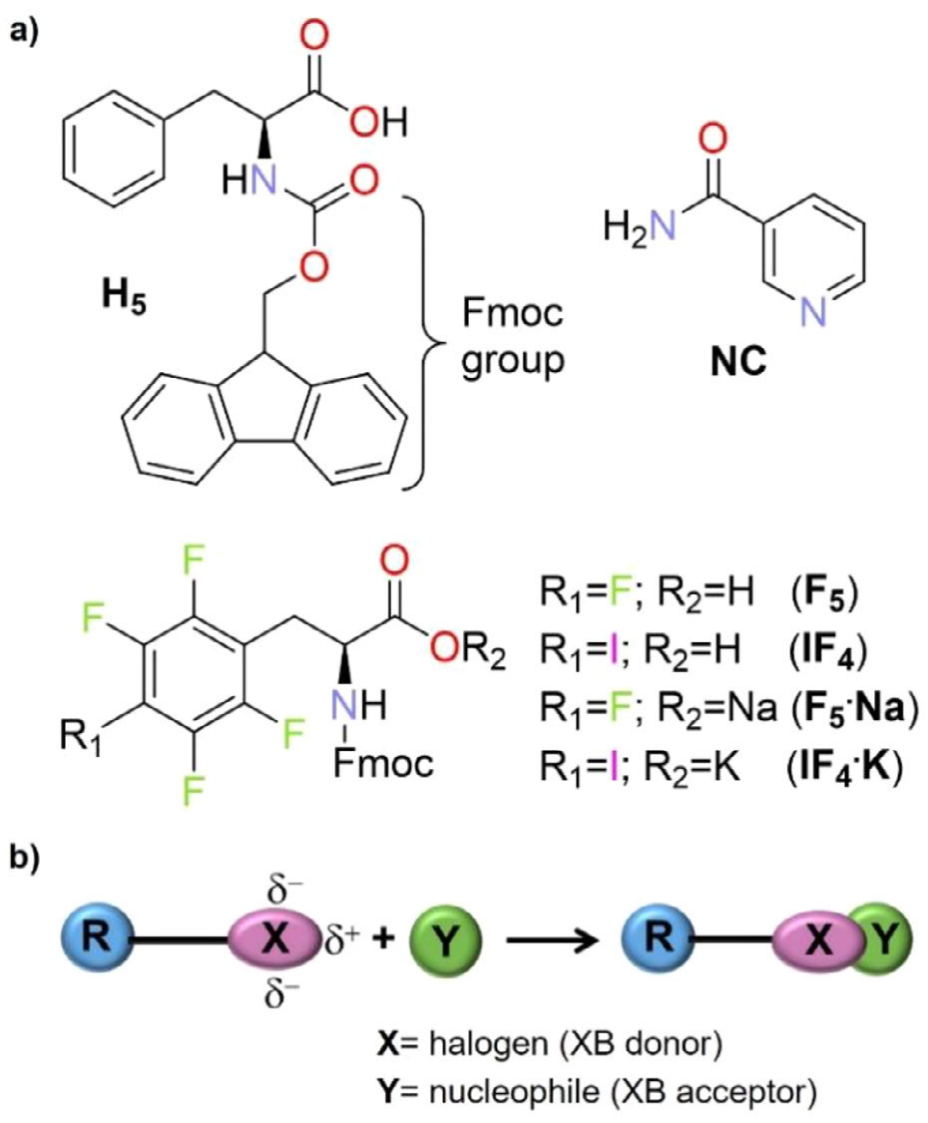
a) Molecular structures of *N*‐Fmoc‐*L*‐phenylalanine (**H_5_**), its halogenated derivatives (**F_5_**, **IF_4_**), their alkali metal salts (**F_5_·Na**, **IF_4_·K**), and nicotinamide/vitamin B_3_ (**NC**). b) Graphical representation of the halogen bond, highlighting the σ‐hole (δ^+^) and the negative potential belt (δ^¯^) on the XB donor.

*N*‐fluorenylmethyloxycarbonyl (Fmoc)‐protected *L*‐phenylalanine (**H_5_**, Figure 1a) is known to be an effective low molecular weight gelator (LMWG) in aqueous solutions. The π‐π stacking of its aromatic groups induces its self‐assembly into ordered fibers, whose further entanglement results in the formation of supramolecular hydrogels.^[^ ^1^, ^2^ ^]^ These 3D networks are generating significant interest for several applications in materials and biomedical sciences, including, for example, tissue engineering, wound healing, and drug delivery.^[^ ^3^, ^4^, ^5^, ^6^, ^7^ ^]^ Halogenation of the **H_5_** phenyl ring has been shown to improve its gelation performance, with the pentafluoro analogue **F_5_** (Figure 1a) forming the most stable and robust hydrogels, even at low concentrations (2 mM, 0.1 wt.%).^[^ ^8^, ^9^ ^]^ We have recently demonstrated that the stiffness and release properties of **F_5_** supramolecular hydrogels can be tailored by exploiting the noncovalent interactions of the gelator with suitable guest molecules.^[^ ^10^ ^]^ In fact, hydrogen bonding (HB) with selected partner molecules was shown to drive the self‐assembly of **F_5_** both in the solid state and in the gel phase. This confirmed the feasibility of a crystal engineering‐based design of multicomponent amino acid hydrogels with tunable strength and properties, useful for the encapsulation and delayed release of bioactive molecules.^[^ ^11^ ^]^

HB is, in fact, only one among many possible noncovalent interactions that can be exploited to drive supramolecular aggregation processes. Another powerful tool is the halogen bond (XB), which is defined as a net attractive interaction between an electrophilic region associated with a halogen atom in a molecular entity (the so‐called σ‐hole), and a nucleophilic region in another, or the same, molecular entity (Figure 1b).^[^ ^12^ ^]^ Steed et al., for instance, demonstrated that halogen bonding between bis(pyridyl urea) and 1,4‐diiodotetrafluorobenzene effectively triggered gelation in organic solvents.^[^ ^13^ ^]^ Iodine is known to be the most efficient XB donor among all halogens and becomes even more electron‐deficient when bound to a polyfluorinated phenyl ring.^[^ ^14^ ^]^ The direct relationship between the positive electrostatic potential of the σ‐hole in Fmoc‐4‐halo‐phenylalanines and the resulting hydrogel strength has previously been demonstrated, with the 4‐iodo‐derivative being the most effective gelator.^[^ ^15^ ^]^

Some of us have also recently demonstrated that unprotected 4‐iodo‐tetrafluoro‐phenylalanine was able to catalyze the synthesis of bis‐(heterocyclic)methanes in water. Experimental evidence showed that the observed catalytic effect was promoted by the XB‐donor ability of its iodine atom.^[^ ^16^ ^]^ We thus wondered whether the combined effect of the fluorination of the phenyl ring and the XB‐donor ability of iodine could further modulate the hydrogelation behavior of a properly designed amino acid, that is, *N*‐Fmoc‐4‐iodo‐tetrafluoro‐phenylalanine (**IF_4_**, Figure 1a).

Starting from these premises, we surmised that the iodine atom in **IF_4_** could establish XB interactions with electron‐rich donor sites in surrounding molecules during gel formation. In particular, it could form significant I···π interactions with Fmoc groups, influence their stacking, and thus modify the fibrillar network.^[^ ^13^ ^]^ We tested the above‐mentioned strategy with respect to a broader range of supramolecular driving forces, aiming to explain and eventually tailor the gelation performance of **IF_4_**. Hydrogels and crystal structures of **IF_4_** were studied under conditions that mimicked those of biological environments. The results were compared with **F_5_**, which was selected as a model effective LMWG with negligible XB‐donor ability.

## 2 Results and Discussion

We initially investigated the hydrogelation behavior of **IF_4_** in ultrapure water in order to compare it with what we previously reported for **F_5_**.^[^ ^10^ ^]^ Unlike **F_5_**, dissolving **IF_4_** in water required heating. However, upon cooling, its solutions underwent almost immediate precipitation (Figure S1). This phenomenon is likely due to the poor water solubility of the iodinated Fmoc‐amino acid. A possible strategy to overcome this obstacle could be the use of a slightly alkaline solvent, which shifts the equilibrium toward the deprotonated carboxylic group of the amino acid. We therefore switched to a 10 mM phosphate‐buffered saline solution (PBS‐Cl, pH = 7.4), containing 2.7 mM potassium chloride and 137 mM sodium chloride, which also mimics the pH and ionic strength of physiological fluids. At physiological pH, both carboxylic groups of **F_5_** and **IF_4_** are deprotonated and may coordinate Na^+^ and K^+^ ions, which are the most abundant metal cations in biological environments.^[^ ^17^ ^]^ It is worth noting that metal cations have been reported to induce hydrogelation of amino acids through metal‐ligand interactions,^[^ ^18^ ^]^ and to influence viscoelastic properties of oligopeptide‐based hydrogels by disrupting the water layer around fibers.^[^ ^19^ ^]^ Understanding the self‐assembly modes of **F_5_** and **IF_4_** metal carboxylates would thus benefit potential bio‐related applications of the resulting supramolecular architectures.

**Figure 2:**
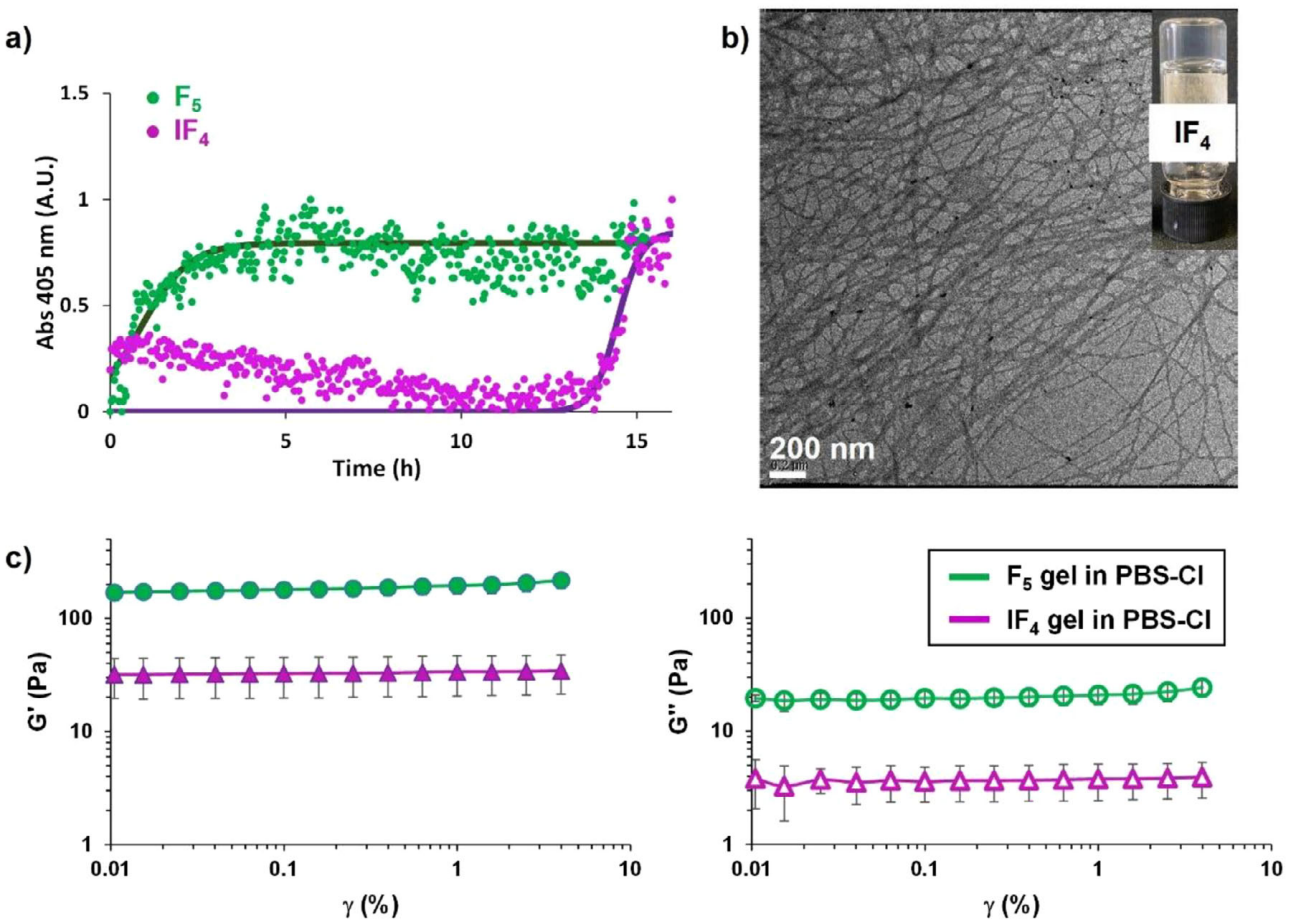
a) Fibril formation kinetics of 0.1 mM solutions of **F_5_** and **IF_4_** in PBS‐Cl. Fittings of experimental data are shown as solid lines. b) TEM image and picture (inset) of **IF_4_** hydrogel (2.5 mM, 5% DMSO in PBS‐Cl). c) Strain sweep experiments (linear viscoelastic region) for **F_5_** (green) and **IF_4_** (purple) hydrogels (2.5 mM, 5% DMSO in PBS‐Cl). Oscillation amplitude table: frequency 0.95 Hz, 5 samples per decade.

Fibril formation kinetics were monitored via turbidimetry, measuring changes in UV‐visible absorbance at 405 nm over time, as previously reported for diphenylalanines.^[^ ^20^ ^]^ Diluted solutions of each amino acid (0.1 mM, in PBS containing 5% v/v DMSO) were incubated under controlled conditions of temperature and stirring (25 °C, 432 rpm). Fibril formation for **IF_4_** was delayed by about 14 hours compared to **F_5_**, even though its fibril growth rate was nearly doubled, with an apparent rate constant K_app_ of 2.94 versus 1.45 h^−1^ (Figures 2a, S2, and Table S4).

Hydrogels were then prepared using the solvent switch method, diluting 100 mM amino acid solutions in DMSO with PBS‐Cl to reach a final gelator concentration of 2.5 mM (0.12 wt.% for **F_5_**, and 0.15 wt.% for **IF_4_**).^[^ ^21^ ^]^ Heating at 100 °C for 1 minute ensured complete dissolution of the amino acids and favored the formation of homogeneous fibrillar networks in both cases, exhibiting similar fiber cross‐sections, as confirmed by Transmission Electron Microscopy (TEM) images (Figures 2b, S3, and S4). However, rheological analyses of the resulting hydrogels were clearly different. Strain sweep experiments performed 24 hours after gel preparation (Figure 2c), showed an almost sevenfold decrease in hydrogel strength for the iodinated LMWG, while **F_5_** in PBS‐Cl gave a storage modulus (G’) comparable to that previously reported in water.^[^ ^10^ ^]^ The use of PBS‐Cl enhanced the solubility of **IF_4_**, allowing gel formation. At the same time, the XB‐accepting ability of chloride ions in the buffer may have altered the fibrillar network by interacting with the XB‐donating iodine atom in **IF_4_**. This additional interaction may be responsible for the observed slower gelation kinetics and reduced hydrogel stiffness, relative to **F_5_**. Scanning Electron Microscopy combined with Energy Dispersive X‐ray Spectroscopy (SEM‐EDS) analyses were performed on hydrogels of both amino acids deposited over carbon tape, air‐dried, and then washed three times with ultrapure water to remove residual buffer salts.

**Figure 3:**
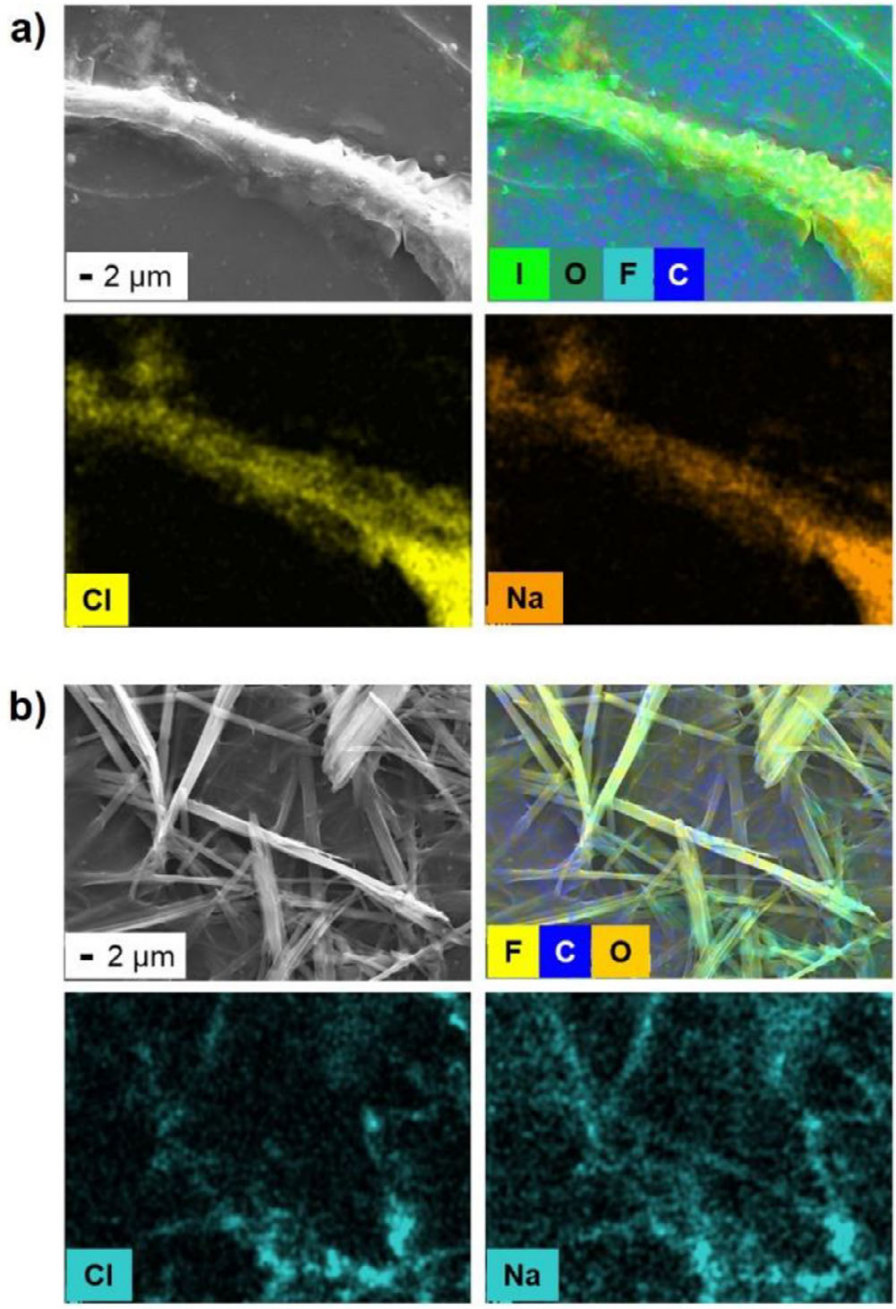
a) Strain SEM and EDS images of **IF_4_** air‐dried hydrogel in PBS‐Cl, after washing with water. Iodine, chlorine, and sodium channels are reported; b) Strain SEM and EDS images of **F_5_** air‐dried hydrogel in PBS‐Cl, after washing with water. Fluorine, chlorine, and sodium channels are reported.

Under these conditions, **IF_4_** fibers appeared larger than **F_5_**, likely due to retention of a greater amount of chloride ions, even after washing, as shown by surface elemental analysis (Figure 3). This result supports our hypothesis of halogen bonding between iodine atoms and chloride ions, suggesting that chlorides influence **IF_4_** aggregation and weaken the final gel, unlike in the case of **F_5_**.

To gain insight into the self‐assembly modes of both amino acids at the molecular level, we then focused on the study of noncovalent interactions involved in their solid‐state packing. Crystallizing the free acid form of *N*‐Fmoc‐protected fluorinated amino acids is extremely challenging, as confirmed by the fact that the only reported crystal structure of **F_5_** was reconstructed from powder X‐ray diffraction data,^[^ ^22^ ^]^ while for **IF_4_** no crystal structure has ever been obtained. Our previous work on **F_5_** demonstrated that the engagement of its carboxylic group in selective interactions with a suitable co‐crystal former provided a higher degree of order in the molecular packing, and thus favored crystallization.^[^ ^10^ ^]^ To simulate the deprotonation of both amino acids and the presence of sodium and potassium cations in the buffer, herein we decided to prepare and crystallize their alkali metal carboxylate salts (**F_5_·Na** and **IF_4_·K**, Figure 1a).

Synthesizing **F_5_·Na** and **IF_4_·K** in alkaline solutions was not feasible, because Fmoc is a base‐labile protecting group. Amino acids’ salts were obtained by mechanochemical synthesis, grinding them with an equimolar amount of sodium or potassium hydroxide pellets. The resulting powders were dissolved in acetone. Slow evaporation at room temperature successfully afforded single crystals of **F_5_·Na** and **IF_4_·K**, within one or two months, respectively. Melting points, powder X‐ray diffraction (PXRD), and Fourier‐transform infrared (FT‐IR) spectra of milled powders and single crystals coincided for both amino acid salts (Figures S5, S6 and S11).^[^ ^23^ ^]^

**Figure 4:**
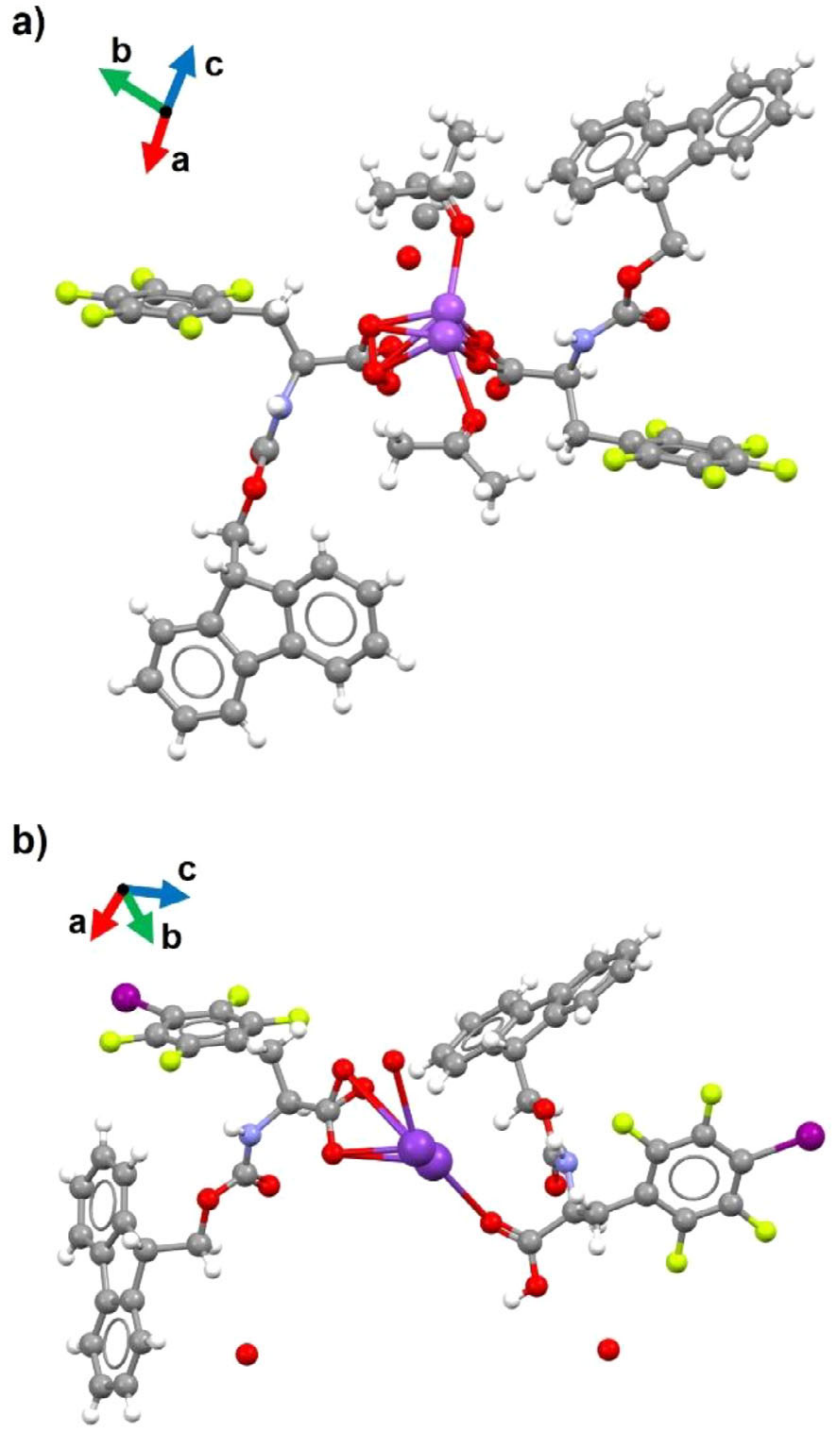
Ball‐and‐stick crystallographic representations of the asymmetric units of: a) **F_5_·Na**; b) **IF_4_·K**. Color code: carbon, gray; oxygen, red; nitrogen, light blue; fluorine, yellow; iodine, purple; sodium and potassium, violet. Metal ions are disordered and split over two positions.

**F_5_·Na** and **IF_4_·K** crystallized in the P1 and P2_1_ space groups, respectively. Both structures displayed two amino acid residues in the asymmetric unit, coordinating one metal cation through the oxygen atoms of their carboxylate or carboxylic groups, revealing a partial salification of the starting amino acids (Figure 4). The main difference between the two asymmetric units is the peculiar spatial orientation of the halogenated phenyl rings in **IF_4_·K**, which did not lie in parallel planes, as instead observed for pentafluorophenyl rings in **F_5_·Na**.

In both cases, metal cations were arranged into infinite rows with amino acid molecules coordinated through carboxylate‐metal interactions. In each 1D chain, amino acid residues stack into two infinite piles on opposite sides of the metal ion. The packing of each pillar was driven by N‐H···O HBs involving either two close carbamate groups, or one carbamate N‐H unit and the C‐terminal oxygen of an adjacent amino acid molecule (Figure S7). Further π‐π interactions between stacked Fmoc units and halogenated phenyl rings strengthened this arrangement in both crystal structures.

**Figure 5:**
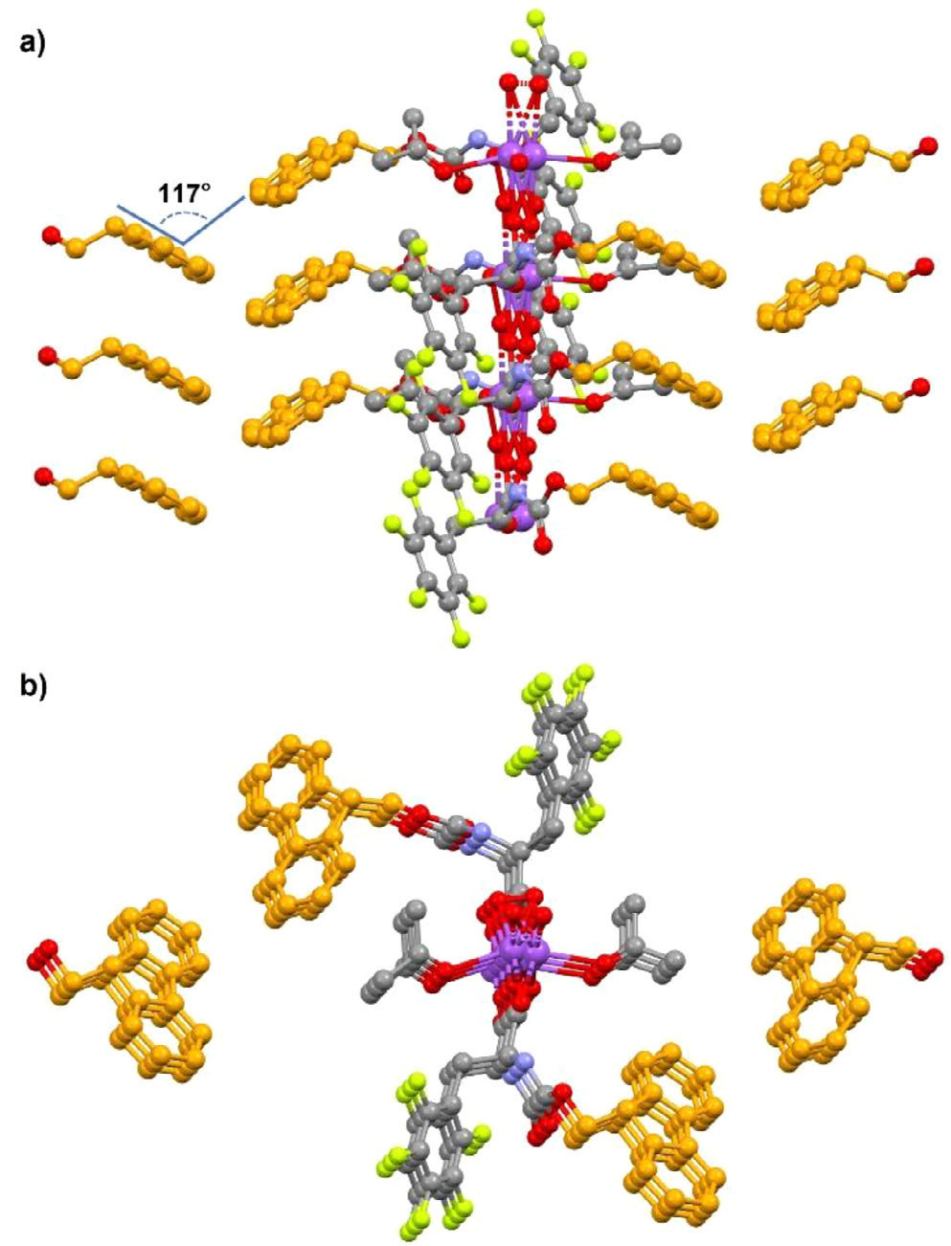
Ball‐and‐stick partial representations of the crystal packing of adjacent amino acid pillars in **F_5_·Na**: a) lateral view; b) top view. For graphical clarity, hydrogen atoms are omitted, and Fmoc aromatic moieties are highlighted in dark yellow. Color code: carbon, gray; oxygen, red; nitrogen, light blue; fluorine, yellow; sodium, violet.

**Figure 6:**
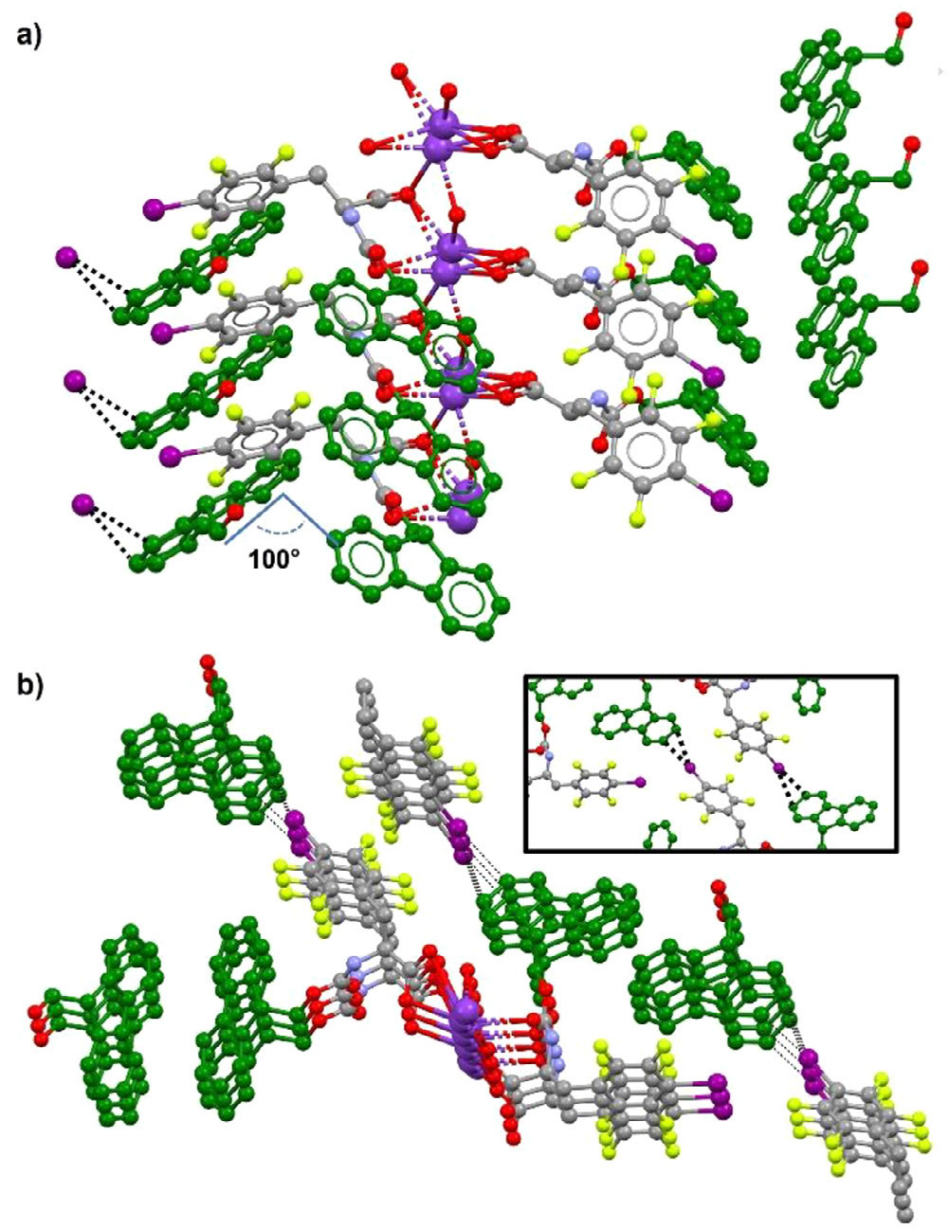
Ball‐and‐stick partial representations of the crystal packing of adjacent amino acid pillars in **IF_4_·K**: a) lateral view; b) top view; inset: I^…^π XB interactions between iodine atoms and Fmoc aromatic rings. For graphical clarity, hydrogen atoms are omitted and Fmoc aromatic moieties are highlighted in green. Color code: carbon, gray; oxygen, red; nitrogen, light blue; fluorine, yellow; iodine, purple; potassium, violet.

The different solid‐state assemblies of the two amino acid salts became more evident when comparing the packing between adjacent cation‐centered pillars. In **F_5_·Na,** the above‐mentioned stacked piles of amino acid residues interacted laterally through a network of C‐H···F and C‐H···π contacts involving Fmoc moieties and perfluorinated rings. Facing Fmoc moieties belonging to adjacent piles formed an angle of 117°, calculated considering the planes of their fluorene rings (Figure 5). Conversely, the inter‐pillar packing in **IF_4_·K** was largely affected by the more complex set of additional interactions established by its iodine atoms with the surrounding chemical environment (Figure 6). In particular, as detailed in the inset of Figure 6b, the σ‐hole of each iodine atom formed a short I···π XB interaction with the closest Fmoc group (I002⋯Centroid_C01L‐CO1G_ distance of 3.392 Å, C01N‐I002⋯ Centroid_C01L‐CO1G_ angle of 171.52°). The steric hindrance of iodine, larger than fluorine, and the above‐cited XB affected the angle between the planes of adjacent fluorene piles, that packed more tightly with an almost orthogonal orientation. As a result, the aromatic rings of facing Fmoc piles involved in XB with iodine formed an angle of 100.07° (Figure 6a). Overall, the more compact arrangement of stacked Fmoc units in the crystals of **IF_4_·K** may perturb, and even adversely affect, its self‐assembly into fibrils. The different packing mode could thus be related to a higher tendency toward crystallization, instead of fibril formation, of the iodinated amino acid. Previous computational studies, performed on simpler model systems, reported a MP2/aug‐cc‐pVTZ interaction energy of ‐0.78 kcal/mol for a hexafluorobenzene dimer,^[^ ^24^ ^]^ and of ‐3.80 kcal/mol for an XB iodobenzene‐benzene dimer.^[^ ^25^ ^]^ The former stabilization effect was given by F···F interactions analogous to those between pentafluorophenyl units in **F_5_·Na**, while the latter was due to iodine···π XBs similar to those observed in **IF_4_·K**. The almost fivefold increase in stabilization energy due to XB could reasonably explain **IF_4_** higher propensity to form crystals, compared to **F_5_**. This could explain why in water such amino acids precipitated without forming hydrogels. In PBS‐Cl, the increased solubility of **IF_4_** and the presence of XB‐accepting anions (like chlorides) could disrupt I···π interactions with the Fmoc units, favoring its self‐assembly into fibers.

A good overlap between FT‐IR spectra and PXRD patterns of mechanically ground powders and lyophilized gels was observed (Figures S6 and S11), confirming the presence of similar driving interactions both in the solid state and in the gel phase. Given this correspondence, the iodine‐induced modification of the solid‐state packing observed in the crystal structure of **IF_4_·K** seemed to be in accordance with the experimental results obtained from rheology in PBS‐Cl. Chloride anions in the buffer behaved as better XB acceptors than Fmoc groups, thanks to their negative charge. As a result, I···Cl¯ interactions could replace weaker I···π contacts among **IF_4_** molecules, favoring Fmoc···Fmoc pairing and thus hydrogelation. To further verify this deduction, we decided to add more effective XB acceptors – such as iodide anions or pyridine‐containing substrates – that were expected to further strengthen the iodinated hydrogel, while affecting only slightly the **F_5_** one. The lower hydration energy of iodide ion compared to chloride (275 vs. 340 kcal/mol), indeed, is known to improve its XB‐acceptor ability in aqueous environments, limiting HB with water molecules.^[^ ^26^ ^]^ For the same reason, the pyridine lone pair could be more available than the chloride ones.

**Figure 7:**
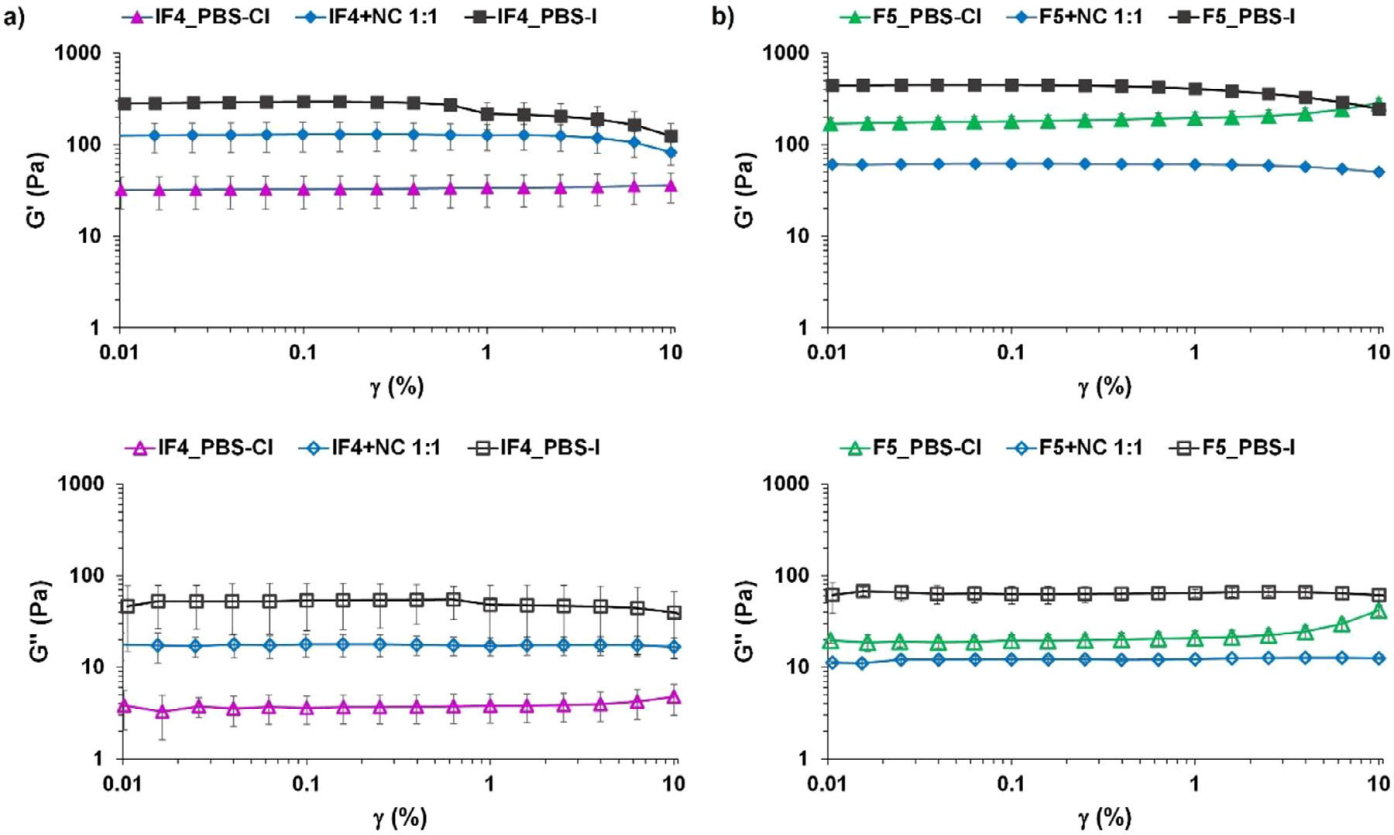
Strain sweep experiments (linear viscoelastic region) for: a) **IF_4_** hydrogels (2.5 mM, 5% DMSO) in PBS‐Cl (purple), in PBS‐Cl with equimolar nicotinamide (blue), and in PBS‐I (black); b) **F_5_** hydrogels (2.5 mM, 5% DMSO) in PBS‐Cl (green), in PBS‐Cl with equimolar nicotinamide (blue), and in PBS‐I (black). Oscillation amplitude table: frequency 0.95 Hz, 5 samples per decade.

First, we prepared a phosphate buffer containing sodium and potassium iodides instead of chlorides (PBS‐I), and used it as a solvent for gelation. An almost 10‐fold increase in G’ was observed for **IF_4_** (Figure 7a), whereas for **F_5_** the storage modulus was less than doubled compared to PBS‐Cl (Figure 7b). Compared to chloride, iodide is actually a better nucleophile for binding the iodine atom of **IF_4_**, and is also less hydrophilic, pushing the fibers toward a more entangled network to avoid contact with water as much as possible.^[^ ^14^ ^]^ A similar effect on G’ was also recorded in the presence of nicotinamide (NC/Vitamin B_3_, Figure 1a), which contains an XB‐accepting pyridyl group. The related **IF_4_** gel became five times stiffer than in neat PBS‐Cl, while for **F_5_** the addition of NC reduced G’ by almost three times (Figure 7). Such findings confirmed the key role of XB in the formation of amino acid‐based supramolecular hydrogels in physiological media, and suggested also the possibility to exploit it for the recognition or encapsulation of XB‐accepting substrates into gel matrices, spanning from anions to bioactive molecules.

## 3 Conclusion

In summary, we have demonstrated that XB can strongly influence both the solid‐state packing and the rheological properties of hydrogels formed by a tailored halogenated Fmoc‐amino acid in biomimetic environments. Compared to **F_5_** – which is an effective LMWG, but a negligible XB‐donor – the presence of an iodine atom on the aromatic ring of **IF_4_** significantly affected its self‐assembly in crystals. At the same time, it slowed down fibril formation and decreased the stiffness of the resulting gels in chloride‐containing phosphate buffer. As previously observed for HB, experimental results confirmed the correlation between crystalline powders and lyophilized hydrogels of Fmoc‐amino acids, even when XB was involved in their supramolecular assembly. This further supports the feasibility of a crystal engineering‐based design for amino acid hydrogels with tunable strength and properties.

Interestingly, when a strong XB acceptor was added to the gelation medium – such as iodide anions in PBS‐I or a pyridine‐containing molecule like vitamin B_3_ – its interaction with the iodine atom in **IF_4_** resulted in increased gel stiffness. These preliminary findings may foreshadow potential future applications of halogen bond‐responsive amino acid hydrogels in anion sensing or in the encapsulation of bioactive molecules, which are currently under investigation and will be reported elsewhere. Moreover, the key structural role of XB in amino acid self‐assembly also suggests the possibility of exploiting it as a secondary nucleation interaction to further enhance gel strength.

## Supporting Information

Detailed description of materials and methods, full characterization of crystals and hydrogels, and further experimental data that support the findings of this study are available in the Supporting Information file. The authors have cited additional references within the Supporting Information^[^ ^27^, ^28^, ^29^, ^30^, ^31^, ^32^, ^33^, ^34^, ^35^, ^36^, ^37^ ^]^

## Conflict of Interests

The authors declare no conflict of intersts.

## Supporting information



Supporting Information

Supporting Information

## Data Availability

The data that support the findings of this study are available from the corresponding author upon reasonable request.
