# Supplementary material for: Halogen Bond‐Tuning of Responsive Supramolecular Amino Acid Hydrogels
Source: Chemistry. 2025 Aug 17;31(54):e01476. doi: 10.1002/chem.202501476 (PMC12462227; doi:10.1002/chem.202501476)
Supplement: Supplementary file 1 — Supporting Information [file CHEM-31-e01476-s002.pdf]

# Table of Contents


1. Materials and methods 2

    1.1 X-ray crystallography 2

    1.2 Transmission Electron Microscopy (TEM) 2

    1.3 Scanning Electron Microscopy- Energy Dispersive Spectroscopy (SEM-EDS) 2

    1.4 Polarized Light Optical Microscopy (POM) 3

    1.5 Attenuated Total Reflectance Fourier-Transform Infrared Spectroscopy (ATR FT-IR) 3

    1.6 Nuclear Magnetic Resonance (NMR) 3

    1.7 Fibrillation kinetics 3

    1.8 Rheology 3

2. Synthesis of compound **$IF_4$** 4

3. Synthesis and crystallization of **$F_5 \cdot Na$** and **$IF_4 \cdot K$** 7

4. Preparation of hydrogels 7

5. Crystallographic data 8

6. Supplementary figures and tables 11

7. References 18

# 1. Materials and methods

*N*-Fmoc-*L*-pentafluorophenylalanine (**$F_5$**) was purchased from abcr GmbH (Germany). Fmoc-4-iodo-tetrafluoro-phenylalanine (**$IF_4$**) was synthesized following a previously reported procedure,[S1] with slight modifications (see Section 2 for details). Nicotinamide (**NC**), and all the other chemicals and solvents were purchased from Alfa-Aesar, Sigma-Aldrich, Apollo, Fluorochem, and used without any further purification. Ultrapure water (Milli-Q grade) was obtained from a Simplicity® water purification system (Merck Millipore). Phosphate buffer saline solution (PBS, 10 mM, pH 7.4) was prepared adding either 2.7 mM potassium chloride and 137 mM sodium chloride (PBS-Cl), or the same concentrations of the corresponding iodide salts (PBS-I).

## 1.1 X-ray crystallography

Single crystal X-ray diffraction data for **$F_5$·Na** (from acetone) were collected using a XtaLAB Synergy diffractometer equipped with a HyPix detector at 300 K (Oxford Cryosystems 800). Unit cell refinement and data reduction were performed with CrysAlisPro 1.171.41.98a.
Data collections for crystals of **$IF_4$·K** (from acetone) and **$F_5$·Na** (from DMSO) were performed at the XRD1 and XRD2 beamlines of the Elettra Synchrotron, Trieste (Italy).[S2] The crystals were dipped in NHV oil (Jena Bioscience, Jena, Germany) and mounted on the goniometer head with kapton loops (MiTeGen, Ithaca, USA). Complete datasets were collected at 100 K (nitrogen stream supplied through an Oxford Cryostream 700) through the rotating crystal method. Data were acquired using monochromatic wavelength of 0.700 Å on a Pilatus 6M hybrid-pixel area detector (DECTRIS Ltd., Baden-Daettwil, Switzerland). The diffraction data were indexed and integrated using XDS.[S3] Crystals grew as tiny needles, in a triclinic crystal form, sensitive to radiation damage. A complete dataset could not be obtained from a single crystal, therefore two partial wedges have been merged using CCP4-Aimless code.[S4,S5] The structure was solved by the dual space algorithm implemented in the SHELXT code.[S6] Fourier analysis and refinement were performed by the full-matrix least-squares methods based on $F^2$ implemented in SHELXL (Version 2019/3).[S7] The Coot program was used for modeling.[S8] Anisotropic thermal motion refinement have been applied to all atoms. Hydrogen atoms were included at calculated positions with isotropic $U_{factors} = 1.2 \bullet U_{eq}$ or $U_{factors} = 1.5 \bullet U_{eq}$ (for methyl and hydroxyl groups; $U_{eq}$ being the equivalent isotropic thermal factor of the bonded non hydrogen atom). Minor thermal restrains (SIMU) have been used on a mobile DMSO molecule. Metal cations in **$F_5$·Na** (from acetone) and in **$IF_4$·K** (from acetone) were affected by positional disorder, which was properly described using the PART command. Pictures were prepared using Ortep-3, Pymol and CCDC Mercury software.[S9-S11]
Powder X-ray diffraction (PXRD) measurements were run at r.t. on a Bruker D2-Phaser diffractometer equipped with Cu radiation (λ = 1.54184 Å), using Bragg−Brentano geometry.

## 1.2 Trasmission Electron Microscopy (TEM)

For TEM analyses, 5 μL of hydrogel samples were deposited on a carbon-coated copper grid and air-dried overnight. Images were acquired with a Transmission Electron Microscope Philips CM 200 FEG (Field emission gun), 200 kV. For cross-section measurements, the cross sections of 50 different fibrils were measured with ImageJ software and data were fitted according to Gaussian function.

## 1.3 Scanning Electron Microscopy-Energy Dispersive Spectroscopy (SEM-EDS)

For SEM analyses, hydrogels were lyophilized and the solid residues were deposited on steel stubs coated by carbon tape. Samples were gilded and images were acquired with a scanning electron microscope Zeiss EVO 50 EP.
Energy Dispersive Spectroscopy qualitative analyses (EDS) were performed with a Bruker XFlash 6l30 probe (resolution: 123 eV at Mn Kα, 45 eV at C Kα and 53 eV at F Kα).

### 1.4 Polarized Light Optical Microscopy (POM)

POM images were registered with a Leica DM4500P POM system that was equipped with a Canon EOS 60D camera. Melting points were determined using the optical microscope combined with a Linkam Scientific temperature-controlled stage (heating rate: 10 °C/min).

### 1.5 Attenuated Total Reflectance Fourier-Transform Infrared Spectroscopy (ATR FT-IR)

ATR FT-IR spectra were measured by using a Nicolet iS50 FTIR spectrometer equipped with an ATR device. Spectra were collected in the medium IR region (64 scans, 4000–400 $cm^{-1}$). All the spectra were measured with a resolution of ± 2 $cm^{-1}$ and corrected for air background.

### 1.6 Nuclear Magnetic Resonance (NMR)

NMR spectra in solution were recorded on a Bruker Avance III 400 MHz spectrometer. Samples were dissolved in deuterated solvent. For $^{13}$C-NMR experiments, chemical shifts were reported in ppm downfield from the residual carbon solvent resonance as internal reference. For $^{19}$F-NMR, experimental chemical shifts were reported in ppm from trifluoroacetic acid (-75.45 ppm), used as external standard.

### 1.7 Fibrillation kinetics

Measurements were performed on a Tecan Infinite 200 PRO plate reader, employing a fully transparent 96 well-plate as sample holder. The instrument was set at 25 °C and orbital shaking was set at 432 revolutions per minute (rpm). Each well was filled with 150 µL of either **F$_5$** or **IF$_4$** (0.1 mM) in 5 % DMSO in PBS-Cl buffer (pH 7.4). Each sample was measured in triplicate.
Method: (i) shake 30 s; (ii) wait 150 s; (iii) shake 2 s; (iv) measure absorption at 405 nm; (v) repeat stages ii-iv for 16 hours. Absorbance of neat solvent mixture was subtracted from each sample. Each measurement was fitted according to equation 1 (Verhulst logistic function):[S12]

$$Y = y_i \frac{y_f}{1 + e^{-(t-t_0)/\tau}}$$

(eq. 1)

Where: Y = absorbance, being $y_i$ and $y_f$ the initial and final absorbance values, respectively;
$t_0$ = time to 50% of maximal signal or the time of inflection point;
$\tau$ = time constant of fibril growth

From the fitting two parameters were extrapolated to figure out the fibril growth process:
$k_{app} = 1/\tau$ apparent growth rate of fibrils
$T = t_0 - 2\tau$ delay time

### 1.8 Rheology

All rheology tests were performed using a KINEXUS Pro+ rheometer (Malvern Panalytical, UK). Samples were pre-formed and directly transferred on the bottom rheometer plate, 24 hours after gelation. A 20-mm parallel plate geometry was used, with a gap distance of 0.5 mm. Temperature was controlled with a Peltier device and maintained at 25 °C. All the oscillatory measurements were performed within the linear viscoelastic range. Each analysis was repeated at least 3 times, and representative measures are reported.
Time sweep experiments were performed at 25 °C for 12 minutes, at a frequency of 0.95 Hz (6 rad $s^{-1}$) and 0.2 % strain. Strain sweep experiments (Linear Viscoelastic Region) were performed at 0.01 % - 100 % strain with a constant frequency of 0.95 Hz (6 rad $s^{-1}$). Frequency sweeps were performed

over a range of frequencies from 0.01 - 16 Hz (0.06 - 100 rad $s^{-1}$) with a constant shear stress chosen within the linear viscoelastic region.

## 2. Synthesis of compound $IF_4$

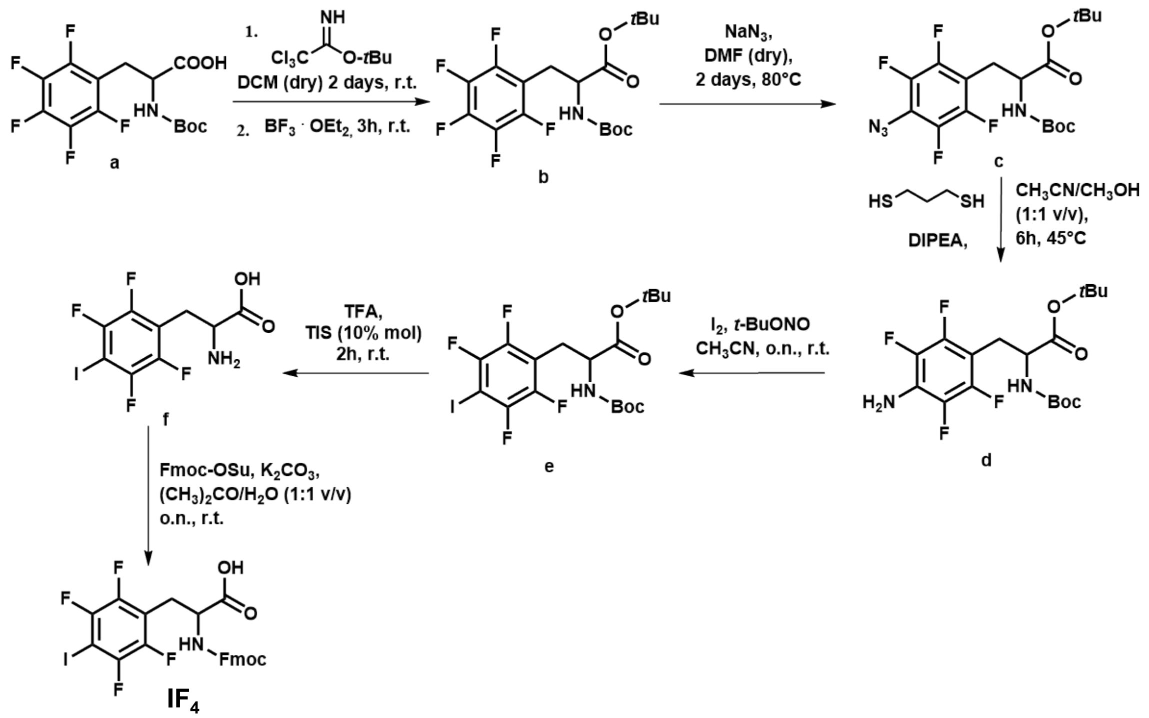


### 2.1 Synthesis of compound b

Commercially available *N*-Boc-pentafluoro-*L*-phenylalanine (**a**, 5 g, 14.1 mmol) was dissolved in dry dichloromethane (DCM) in 0.1 M concentration. After slow addition of *tert*-butyl 2,2,2-trichloroacetimidate (5.03 mL, 0.28 mmol), the mixture was stirred at r.t. for 2 days and monitored by TLC. The formation of a colorless solid occurred. Subsequently, $BF_3 \cdot OEt_2$ was added (260 µL, 2.1 mmol), and the mixture was stirred for further two hours. Then, the reaction was quenched by adding a $NaHCO_3$ saturated aqueous solution, and the resulting mixture was extracted with DCM (3 × 50 mL). The combined organic layers were washed with brine, dried over $Na_2SO_4$, and concentrated under vacuum. The crude product was purified by flash column chromatography (hexane/AcOEt 10:1), yielding 4.2 g of compound **b** (72 % yield) as a colorless oil that solidified upon standing ($R_f$ = 0.3, permanganate staining).

**b:** $^1$H-NMR (400 MHz, $CDCl_3$): δ (ppm) = 5.12 (m, 1H, NH), 4.44 (m, 1H, CHNH), 3.23 (dd, 1H, J = 4.0, 13.5 Hz, PhCHH), 3.04 (dd, J = 8.0, 13.2 Hz, 1H, PhCHH), 1.45 (s, 9H, COOCCH3), 1.39 (s, 9H, NCOOCCH3). $^{19}$F-NMR (376 MHz, $CDCl_3$): δ (ppm) = -141.87 (dd, 2F, J = 22.5, 7.9 Hz, 2F), -156.11 (t, 1F, J = 20.8 Hz), -162.75 (td, 2F, J = 22.1, 23.0, 7.4 Hz).

### 2.2 Synthesis of compound c

In a dried round-bottom flask, compound **b** (4.2 g, 10 mmol) was dissolved in dry DMF in 0.7 M concentration. After complete dissolution, sodium azide (1.33 g, 20 mmol) was added at 0 °C under nitrogen flux. The resulting solution was immersed in a 80 °C pre-heated oil bath, and stirred at the same temperature for two days. The mixture slowly turned brownish. The reaction was then poured into cool water (200 mL). The product precipitated as a brown solid, which was washed with clean water, and then purified by flash column chromatography (hexane/AcOEt 97:3), yielding 3 g of

compound **c** ($R_f$= 0.39, 69% yield) as a colorless powder. From the same column is possible to isolate also compound d as white solid (Rf 0.02 in Hexane/AcOEt 97:3, 3% yield).

**c:** $^1$H-NMR (400 MHz, $CDCl_3$): δ (ppm) = 5.11 (d, J = 8.0 Hz, 1H, NH), 4.44 (d, J = 6.6 Hz, 1H, CHNH), 3.22 (dd, J = 14.1, 5.8 Hz, 1H, PhCHH), 3.03 (dd, J = 14.0, 7.51 Hz, 1H, PhCHH), 1.45 (s, 9H, COOCCH3), 1.40 (s, 9H, NCOOCH3). $^{19}$F-NMR (376 MHz, $CDCl_3$): δ (ppm) = -142.11 (dd, 2F, J = 21.2, 10.1 Hz), -152.55 (dd, 2F, J = 21.2, 10.1 Hz).

### 2.3 Synthesis of compound d

Compound **c** (2.5 g, 5.74 mmol) was dissolved in a 1:1 mixture of MeOH/$CH_3CN$ in 0.15 M concentration. To the resulting mixture, 1,3-propanedithiol (1.15 mL, 11.5 mmol) and *N*,*N*-diisopropylethylamine (2 mL, 11.5 mmol) were added. A slight effervescence was observed. The reaction was immersed in a 45 °C pre-heated oil bath, and stirred for six hours. The mixture was then concentrated, poured into 100 mL of saturated $NH_4Cl$ solution, and extracted with DCM (3 × 30 mL). The combined organic layers were washed with brine, dried over $Na_2SO_4$, and concentrated under vacuum. The resulting waxy brownish solid was purified by flash column chromatography, eluting with hexane/AcOEt 8:2, to give compound **d** as a colorless solid (77 % yield, $R_f$ = 0.43).

**d:** $^1$H-NMR (400 MHz, $CDCl_3$): δ (ppm) = 5.08 (d, J = 8.4 Hz, 1H, NH), 4.41 (q, J = 7.3 Hz, 1H, CHNH), 3.14 (dd, J = 14.4, 5.8 Hz, 1H, PhCHH), 2.97 (dd, J = 14.1, 7.3 Hz, 1H, PhCHH), 1.44 (s, 9H, COOCCH3), 1.40 (s, 9H, NCOOCCH3). $^{19}$F-NMR (376 MHz, $CDCl_3$): δ (ppm) = -145.00 (dd, 2F, J = 20.9, 8.8 Hz), -162.41 (m, 2F).

### 2.4 Synthesis of compound e

To a 0.1 M solution of compound **d** (1.8 g, 4.46 mmol) in freshly distilled $CH_3CN$, cooled to 0 °C and protected from light, iodine (2.26 g, 8.92 mmol) was added. After 15 min, *t*-butyl nitrite (780 µL, 6.69 mmol) was added dropwise over a period of 5 min, at 0°C. The resulting solution was allowed to return at r.t. and stirred for further 2 h. Then, it was concentrated to reduced volume avoiding the use of heat, and poured into 100 mL of a 2 M $NaHSO_3$ solution. The aqueous mixture was extracted with DCM (4 × 120 mL). The collected organic layers were washed with brine, dried over $Na_2SO_4$ and concentrated. The crude was purified via flash column chromatography eluting with a DCM/hexane gradient (from 1:1 to 1:0), yielding a colorless solid in 52 % yield ($R_f$ = 0.21).

**e:** $^1$H-NMR (400 MHz, $CDCl_3$): δ (ppm) = 5.12 (d, J = 8.1 Hz, 1H, NH), 4.47 (m, 1H, CHNH), 3.25 (dd, 1H, J = 4.0, 12.0, PhCHH), 3.07 (ddt, 1H J = 13.7, 7.6, 1.6 Hz, PhCHH), 1.44 (s, 9H, COOCCH3), 1.38 (s, 9H, NCOOCCH3). $^{19}$F-NMR (376 MHz, $CDCl_3$): δ (ppm) = -121.31 (dd, 2F J = 22.0, 9.9 Hz), -140.08 (m, 2F).

### 2.5 Synthesis of compound f

At 0 °C, compound **e** (0.42 g, 0.8 mmol) was dissolved in trifluoroacetic acid (3 mL, 39.2 mmol). Tri-isopropyl-silane (TIPS 10 %, 17 µL, 0.08 mmol) was added to the solution, under stirring. The resulting mixture was allowed to warm up to r.t., and further stirred for 2 hours. Then, toluene (5 mL) was added, and the mixture was evaporated to dryness. The crude was washed with $Et_2O$/hexane (1:1 v/v, 6 × 5 mL). The yellowish oil obtained was washed with clean $Et_2O$, and the resulting colorless precipitate was separated through centrifugation (6000 rpm, 5 min). The solid was washed again with $Et_2O$ (3 × 10 mL), and directly used for the next step ($R_f$ = 0.16 in $CHCl_3$/AcOEt 2:1 + 0.5 $\%_{v/v}$ $CH_3CO_2H$, permanganate staining, strongly fluorescent spot at 365 nm).

**f:** $^1$H-NMR (400 MHz, methanol-$d_4$): δ (ppm) = 3.75 (dd, 1H, J = 7.9, 6.8 Hz, CHNH), 3.42 (m, 1H, PhCHH), 3.19 (m, 1H, PhCHH). $^{19}$F-NMR (376 MHz, methanol-$d_4$): δ (ppm) = -123.79 (m, 2F), -141.91 (m, 2F).

**2.6 Synthesis of $IF_4$**

Compound **f** (0.2 g, 0.55 mmol) and potassium carbonate (0.38 g, 7.7 mmol) were dissolved in a 1:1 acetone/water mixture (0.3 M final concentration of **f**). After cooling the solution to 0 °C, Fmoc-OSu (0.222 g, 0.66 mmol) was added, then it was stirred at r.t. overnight, protected from light. The reaction mixture was concentrated, and added with HCl 6M (3 mL). The resulting precipitate was separated through centrifugation (7000 rpm, 7 min), and washed with hexane (10 × 10 mL) to get the final product as a colorless solid (81 % yield, $R_f$ = 0.15 in hexane/AcOEt 1:1 v/v + 1 % $CH_3CO_2H$).

**IF4:** $^1$H-NMR (400 MHz, acetone-$d_6$): δ (ppm) = 7.85 (d, 2H, J = 7.6 Hz, CH), 7.65 (d, 2H, J = 7.5 Hz, CH), 7.41 (m, 2H, CH), 7.31 (m, 2H, CH), 6.95 (d, 1H, J = 8.9 Hz, COOH), 4.62 (m, 1H CHNH), 4.25 (m, 3H, CH2, OCH2CH), 3.43 (dd, 1H, J = 14.2, 5.7 Hz, PhCHH), 3.32 (dd, 1H, J = 14.1, 8.8 Hz, PhCHH). $^{19}$F-NMR (376 MHz, acetone-$d_6$): δ (ppm) = -125.23 (m, 2F), -143.53 (m, 2F). $^{13}$C-NMR (101 MHz, acetone-$d_6$): δ (ppm) = 171.94 (s, 1C, COOH), 156.80 (s, 1C, NHCOOFmoc), 144.95 (d, 2C, J = 1.7 Hz), 142.09 (d, 2C, J = 2.9 Hz), 128.54 (s, 2C), 127.94 (s, 2C), 126.09 (d, 2C, J = 5.6 Hz), 120.79 (d, 2C, J = 2.1 Hz), 118.39 (s, 1C, CCH2), 67.45 (s, 1C, OCH2), 53.60 (s, 1C, CHNH), 47.93 (s, 1C, OCH2CH), 26.68 (s, 1C, CH2), 26.09 (s, CI). IR (ATR): υ ($cm^{-1}$) = 3345 (m), 2923 (m), 1731 (s), 1698 (s), 1533 (m), 1477 (m), 1266 (m), 1250 (m), 1230 (m), 1086 (m), 1049 (m), 949 (m), 871 (s), 802 (s), 757 (s), 738 (s), 621 (s). MS (ESI+): m/z (%) = 608 (100) $[M+Na]^+$. MS (ESI-): m/z (%) = 584 (100) $[M]^-$.

## 3. Synthesis and crystallization of $F_5$·Na and $IF_4$·K

Compounds **$IF_4$·K** and **$F_5$·Na** were prepared by mechanochemical synthesis, using a Retsch MM400 ball mill with 2.5 mL vessels, operating at 15 Hz.

### 3.1 Synthesis and crystallization of $F_5$·Na

**$F_5$·Na** was obtained through liquid-assisted grinding of **$F_5$** (50 mg, 0.105 mmol) and sodium hydroxide (4.2 mg, 0.105 mmol), upon addition of 20 μL of acetone. Ball milling was performed for 30 min. **$F_5$·Na** milled powder (10 mg) was dissolved in the minimum amount of acetone. Slow evaporation at r.t. afforded colourless single crystals in one month.

**$F_5$·Na:** m.p. 215 °C. FT-IR (selected bands, $cm^{-1}$): 3323, 1680, 1599, 1520, 1500, 1397, 1264, 1123, 1044, 961, 734, 540.

### 3.2 Synthesis and crystallization of $IF_4$·K

**$IF_4$·K** was obtained through liquid-assisted grinding of **$IF_4$** (50 mg, 0.085 mmol) and potassium hydroxide (4.8 mg, 0.085 mmol), upon addition of 150 μL of acetone and 5 μL of water. Ball milling was performed for 25 min. **$IF_4$·K** milled powder (10 mg) was dissolved in the minimum amount of acetone. Slow evaporation at r.t. afforded colourless single crystals in two months.

**$IF_4$·K:** m.p. 223 °C. FT-IR (selected bands, $cm^{-1}$): 3322, 1680, 1594, 1473, 1394, 1253, 1088, 1046, 732, 621, 540.

## 4. Preparation of hydrogels

Following the solvent switch method reported by Nilsson *et al.*,[S13] gelators **$F_5$** and **$IF_4$** (100 mM) were dissolved in DMSO, and the resulting stock solutions were diluted with PBS-Cl or PBS-I up to a final amino acid concentration of 2.5 mM (0.12 wt% for **$F_5$** and 0.15 wt% for **$IF_4$**, respectively). Before dilution, the solvent was heated up to 50 °C, in order to help solubilization. Diluted samples were then vortexed for 3 seconds, heated at 100 °C for 1 minute to allow complete dissolution of amino acids, and then slowly cooled down to r.t. (1 °C/min cooling rate). Gelation occurred after overnight rest. Multicomponent hydrogels were obtained with the same procedure, diluting **$F_5$+NC** and **$IF_4$+NC** stock solutions (in 1:1 molar ratio).

## 5. Crystallographic data

**Table S1.** Crystallographic data and refinement details for crystal **$F_5$·Na** obtained by slow evaporation of **$F_5$·Na** milled powder (10 mg) dissolved in the minimum amount of acetone.

| | **$F_5$·Na** |
|---|---|
| CCDC Number | 2372161 |
| Chemical Formula | $C_{52.5}H_{39}F_{10}N_2NaO_{9.5}$ |
| Formula weight | 1062.85g/mol |
| Temperature | 300.85(18)K |
| Wavelength | 1.54184Å |
| Crystal system | Triclinic |
| Space Group | *P* 1 |
| Unit cell dimensions | $a$ = 5.3997(2)Å |
| | $b$ = 14.5936(4)Å |
| | $c$ = 17.7150(5)Å |
| | $\alpha$ = 69.255(2)° |
| | $\beta$ = 86.239(2)° |
| | $\gamma$ = 82.783(2)° |
| Volume | 1294.78(7)$Å^3$ |
| Z | 1 |
| Density (calculated) | 1.363 $g{\cdot}cm^{-3}$ |
| Absorption coefficient | 1.089 $mm^{-1}$ |
| F(000) | 545 |
| Theta range for data collection | from 6.83° to 152.82° |
| Index ranges | $-6 \leq h \leq 6$, $-18 \leq k \leq 16$, $-22 \leq l \leq 21$ |
| Resolution | 0.63 Å |
| Reflections collected | 15458 |
| Independent reflections | 6515 [$R_{int}$ = 0.0468, $R_{sigma}$ = 0.0530] |
| Refinement method | Full-matrix least-squares on $F^2$ |
| Data / restraints / parameters | 6515/90/781 |
| Goodness-of-fit on $F^2$ | 1.038 |
| Final R indices [I>2σ I)] | $R_1$ = 0.0531, $wR_2$ = 0.1392 |
| R indices (all data) | $R_1$ = 0.0605, $wR_2$ = 0.1486 |
| Largest diff. peak and hole | 0.58 and -0.20 $eÅ^{-3}$ |
| Absolute structure parameter | 0.04(13) |

$R_1 = \Sigma \,||Fo|–|Fc|| \,/\, \Sigma\, |Fo|$, $wR_2 = \{\Sigma\, [w(Fo^2 – Fc^2)^2] \,/\, \Sigma\, [w(Fo^2)^2]\}^{½}$

**Table S2.** Crystallographic data and refinement details for crystal **$IF_4$·K** obtained by slow evaporation of **$IF_4$·K** milled powder (10 mg) dissolved in the minimum amount of acetone.

| | **$IF_4$·K** |
|---|---|
| CCDC Number | 2372162 |
| Chemical Formula | $C_{24}H_{17}F_4IK_{0.5}NO_{5.5}$ + [Solvent] |
| Formula weight | 629.83 g/mol |
| Temperature | 100(2) K |
| Wavelength | 0.700 Å |
| Crystal system | Monoclinic |
| Space Group | $P\,2_1$ |
| Unit cell dimensions | $a$ = 23.094(5) (2) Å |
| | $b$ = 4.9780(10)Å |
| | $c$ = 23.760(5) |
| | $\alpha$ = 90° |
| | $\beta$ = 111.08(3)° |
| | $\gamma$ = 90° |
| Volume | 2548.6(10)Å$^3$ |
| Z | 4 |
| Density (calculated) | 1.643g·cm$^{-3}$ |
| Absorption coefficient | 1.332mm$^{-1}$ |
| F(000) | 1244.0 |
| Theta range for data collection | from 1.81° to 59.23° |
| Index ranges | $-32 \leq h \leq 32$, $-6 \leq k \leq 6$, $-33 \leq l \leq 33$ |
| Resolution | 0.706 Å |
| Reflections collected | 46716 |
| Independent reflections | 14611[$R_{int}$ = 0.0378, $R_{sigma}$ = 0.0358] |
| Refinement method | Full-matrix least-squares on $F^2$ |
| Data / restraints / parameters | 14611/15/681 |
| Goodness-of-fit on $F^2$ | 1.053 |
| Final R indices [I>2σ(I)] | $R_1$ = 0.0330, $wR_2$ = 0.0967 |
| R indices (all data) | $R_1$ = 0.0342, $wR_2$ = 0.0977 |
| Largest diff. peak and hole | 1.11 and -1.13 eÅ$^{-3}$ |
| Absolute structure parameter | 0.001(6) |

We are unable to model the residual electron density likely related to disordered solvent molecule. This was treated using a solvent mask (Program SQUEEZE).[S14]

**Table S3.** Crystallographic data and refinement details for crystal **$F_5$·Na** obtained by slow evaporation of **$F_5$·Na** milled powder (10 mg) dissolved in the minimum amount of DMSO.

| | **$F_5$·Na (from DMSO)** |
|---|---|
| CCDC Number | 2354251 |
| Chemical Formula | $(C_{24}H_{16}F_5NO_4)[C_{24}H_{15}F_5NO_4]Na(C_2H_6O)_2$ |
| Formula weight | 1132.99 g/mol |
| Temperature | 100(2) K |
| Wavelength | 0.700 Å |
| Crystal system | Triclinic |
| Space Group | *P* 1 |
| Unit cell dimensions | $a$ = 10.663(2) Å |
| | $b$ = 14.723(3) Å |
| | $c$ = 17.591(4) Å |
| | $\alpha$ = 109.56(3)° |
| | $\beta$ = 92.38(3)° |
| | $\gamma$ = 104.14(3)° |
| Volume | 2500.4(10) $Å^3$ |
| Z | 2 |
| Density (calculated) | 1.505 $g·cm^{-3}$ |
| Absorption coefficient | 0.204 $mm^{-1}$ |
| F(000) | 1164 |
| Theta range for data collection | from 2.442 to 54.84° |
| Index ranges | $-13 \leq h \leq 13$, $-19 \leq k \leq 17$, $-0 \leq l \leq 23$ |
| Resolution | 0.76 Å |
| Reflections collected | 31829 |
| Independent reflections | 11434 [$R_{int}$ = 0.0759, $R_{sigma}$ = 0.0696] |
| Refinement method | Full-matrix least-squares on $F^2$ |
| Data / restraints / parameters | 11440/ 9 / 1397 |
| Goodness-of-fit on $F^2$ | 1.047 |
| Final R indices [I>2σ(I)] | $R_1$ = 0.0576, $wR_2$ = 0.1331 |
| R indices (all data) | $R_1$ = 0.0831, $wR_2$ = 0.1494 |
| Largest diff. peak and hole | 0.54 and -0.79 $eÅ^{-3}$ |
| Absolute structure parameter | 0.19(10) |

$R_1 = \Sigma \,||Fo|–|Fc|| / \Sigma\, |Fo|$, $wR_2 = \{\Sigma\, [w(Fo^2 – Fc^2)^2] / \Sigma\, [w(Fo^2)^2]\}^{½}$

**Table S4.** Geometrical parameters of notable contacts found in **$F_5$·Na** crystal packing from DMSO.

| **D···A** | **d(D-H) (Å)** | **d(H···A) (Å)** | **d(D···A) (Å)** | **<(DHA) (°)** |
|---|---|---|---|---|
| O1_1-H1_1···O1_2 | 0.84 | 1.68 | 2.517(5) | 175.9 |
| N1_1-H1A_1···O3_4#1 | 0.88 | 2.20 | 3.032(5) | 157.0 |
| N1_2-H1A_2···O2_3 | 0.88 | 2.13 | 2.993(5) | 167.1 |
| N1_3-H1A_3···O2_2#1 | 0.88 | 2.15 | 3.013(5) | 166.6 |
| O1_4-H1_4···O1_3#2 | 0.84 | 1.67 | 2.513(5) | 176.7 |
| N1_4-H1A_4···O3_1 | 0.88 | 2.39 | 3.215(5) | 156.5 |

Symmetry transformations used to generate equivalent atoms: #1: x+1,y,z; #2: x-1,y,z

## 6. Supplementary figures and tables

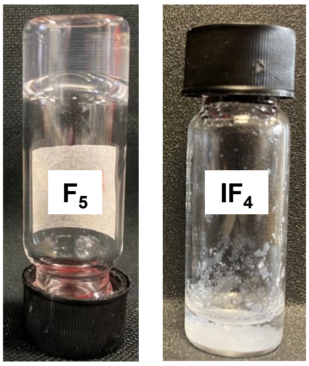


**Figure S1.** Pictures showing the different behaviour of $\mathbf{F_5}$ and $\mathbf{IF_4}$ in ultrapure water at 2.5 mM concentration: the former formed a stable transparent hydrogel (left), whereas the latter precipitated almost immediately (right).

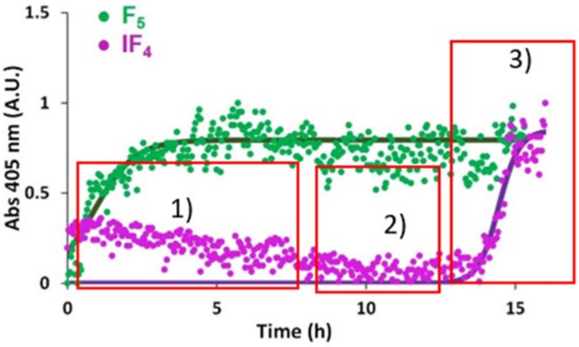


**Figure S2.** Fibril formation kinetics of 0.1 mM solutions of $\mathbf{F_5}$ and $\mathbf{IF_4}$ in PBS-Cl. Red rectangles highlight the different steps for $\mathbf{IF_4}$: 1) the diluted solution of $\mathbf{IF_4}$ in PBS-Cl buffer showed an initial absorbance higher than zero, as a consequence of the aggregation of amino acid molecules, with iodine atoms staying close to Fmoc groups and far from water; 2) after about 10 hours of constant stirring, such aggregates started to disrupt, favouring hydration of $\mathbf{IF_4}$ molecules, and the absorbance became zero; 3) the formation of fibrils started and took place very fast, thanks to XB interactions between iodine atoms of $\mathbf{IF_4}$ and chloride anions in the buffer.

**Table S5.** Apparent growth rates of fibrils ($K_{app}$) and fibril formation delay time measured in PBS-Cl for $\mathbf{F_5}$ and $\mathbf{IF_4}$ (0.1 mM).

| | $K_{app}$ ($h^{-1}$) | Delay (h) |
|---|---|---|
| $\mathbf{F_5}$ | 1.45 | 0 |
| $\mathbf{IF_4}$ | 2.94 | 13.8 |

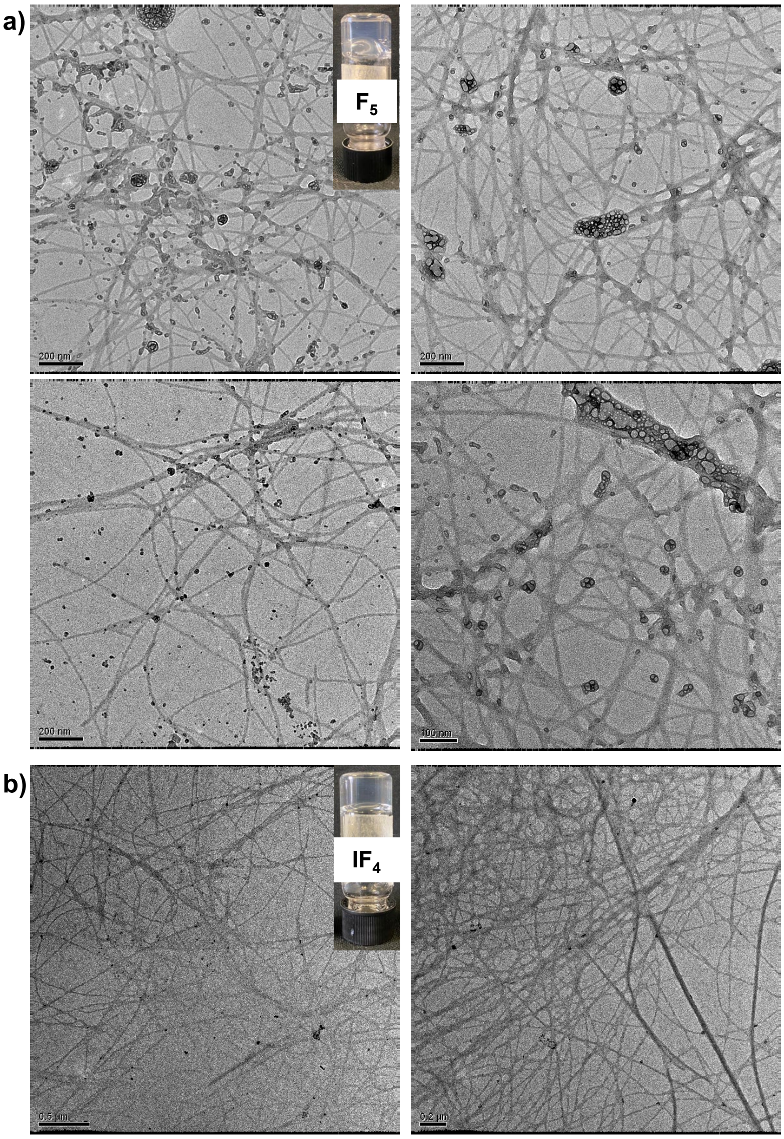


**Figure S3.** TEM images and picture (inset) of: a) **$F_5$** hydrogel in PBS-Cl (2.5 mM); b) **$IF_4$** hydrogel in PBS-Cl (2.5 mM).

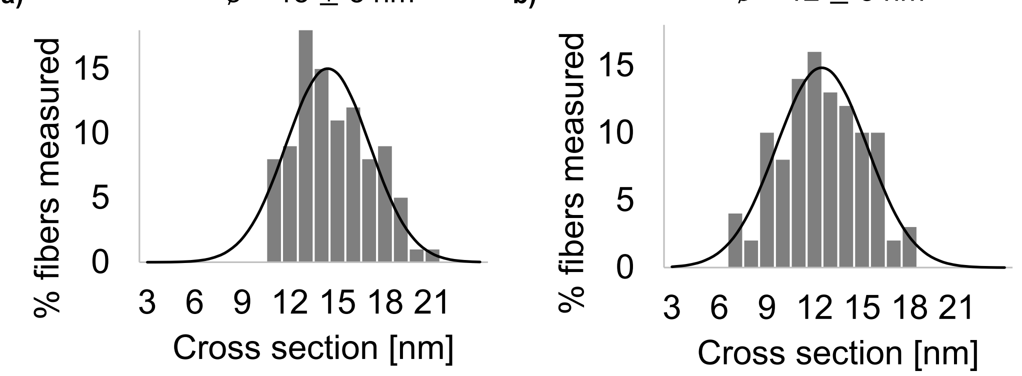


**Figure S4.** Statistical analyses of fibril cross sections, as measured from TEM images on 50 different fibrils in PBS-Cl: a) **$F_5$** hydrogel; b) **$IF_4$** hydrogel. Gaussian fittings of data distributions are shown as solid black lines.

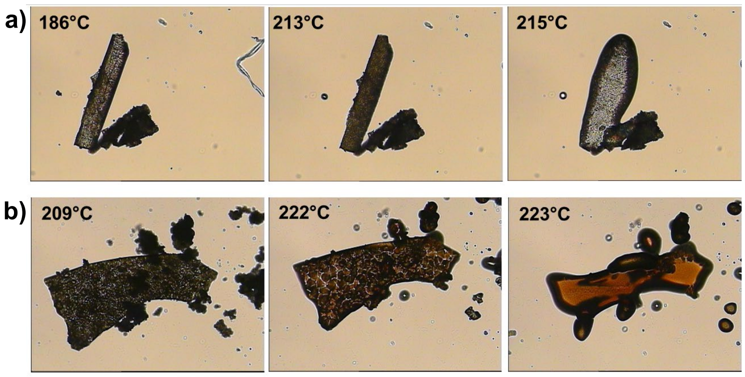


| | | m.p. (°C) |
|---|---|---|
| **$F_5$·Na** | Single crystals | 215 |
| | Milled powder | 213 |
| **$IF_4$·K** | Single crystals | 223 |
| | Milled powder | 220 |

**Figure S5.** POM images showing the melting transitions for single crystals of: a) **$F_5$·Na**; b) **$IF_4$·K**. Comparative chart of melting temperatures for single crystals and ball-milled powders of **$F_5$·Na** and **$IF_4$·K**.

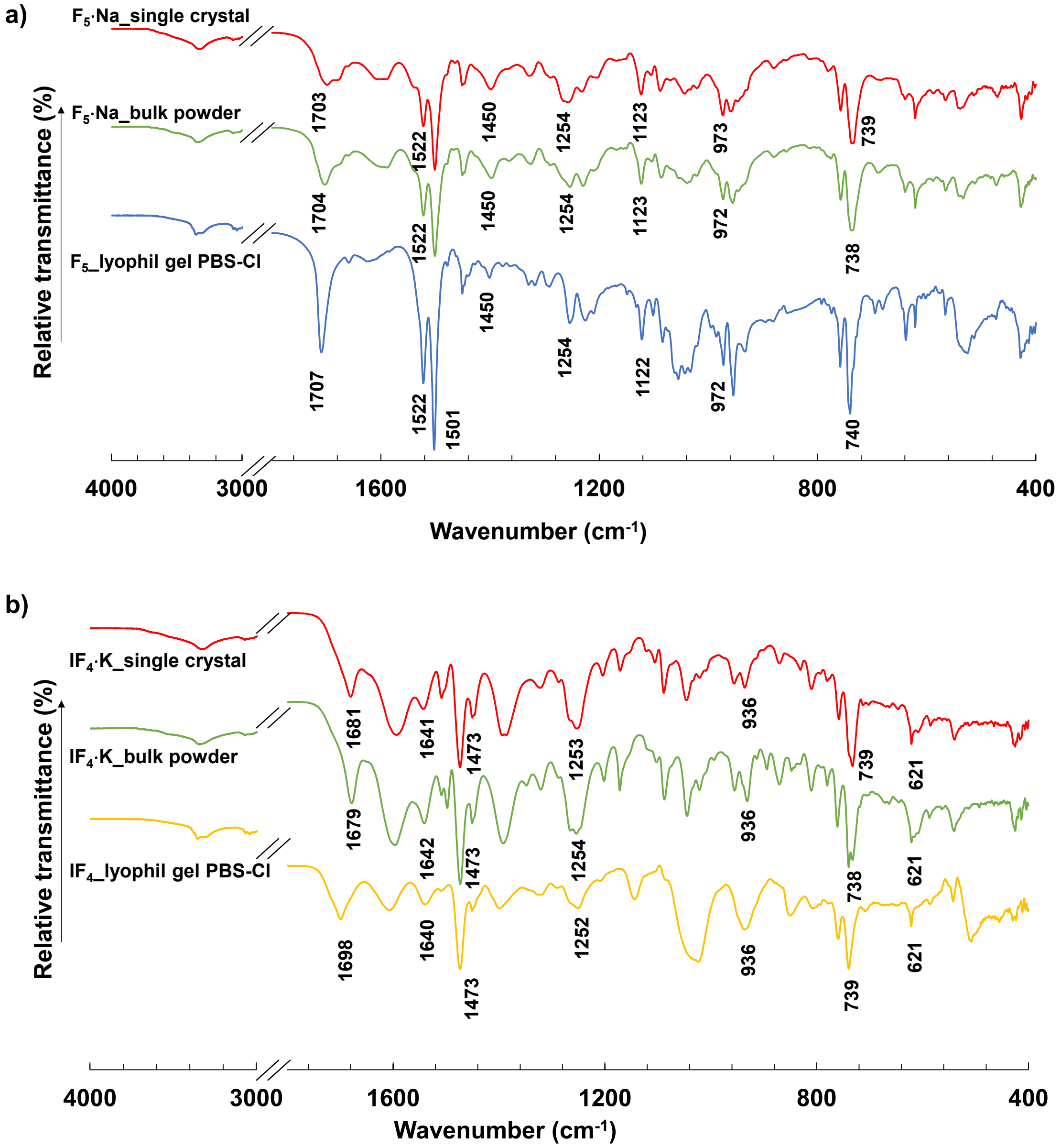


**Figure S6.** FT-IR spectra of: a) **$F_5$·Na** single crystals (red), milled powder (green), and lyophilized hydrogel from PBS-Cl (blue); b) **$IF_4$·K** single crystals (red), milled powder (green), and lyophilized hydrogel from PBS-Cl (yellow).

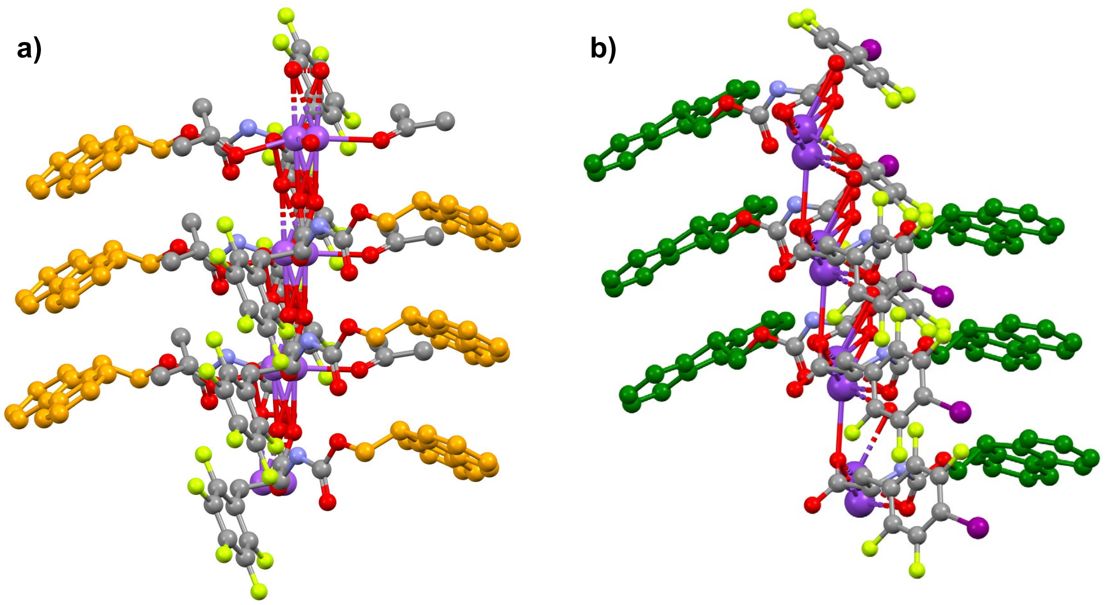


**Figure S7.** Ball-and-stick view of stacked amino acid residues coordinated to one metal cations row for: a) **$F_5$·Na**; b) **$IF_4$·K**. Stacked pillars of Fmoc groups are highlighted in dark yellow and green, respectively. Hydrogen atoms are omitted for clarity. Color code: carbon, gray; oxygen, red; nitrogen, light blue; fluorine, yellow; iodine, purple; sodium and potassium, violet.

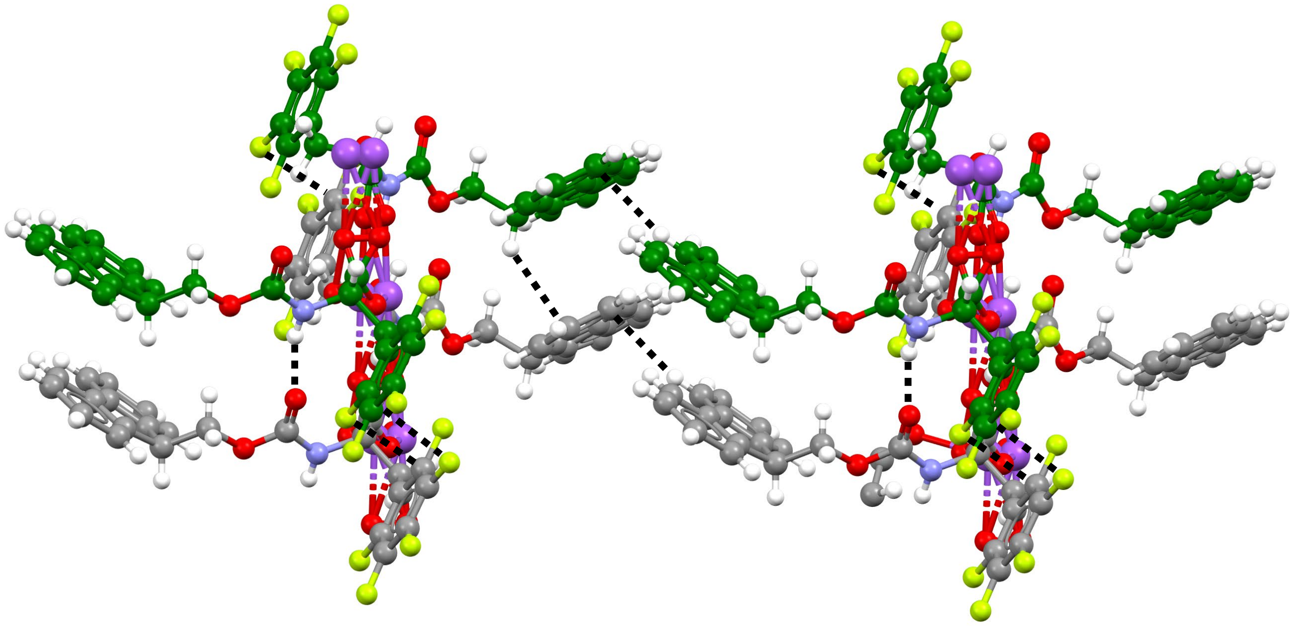

**Figure S8.** Ball-and-stick partial view of the crystal packing of neighboring **$F_5$·Na** residues placed on the same side of a metal ions row. N-H···O HBs involving either two close carbamate groups, or one carbamate N-H unit and the C-term oxygen of an adjacent amino acid molecule, are indicated by black dotted lines. Color code: carbon, gray; oxygen, red; nitrogen, light blue; fluorine, yellow; sodium and potassium, violet.

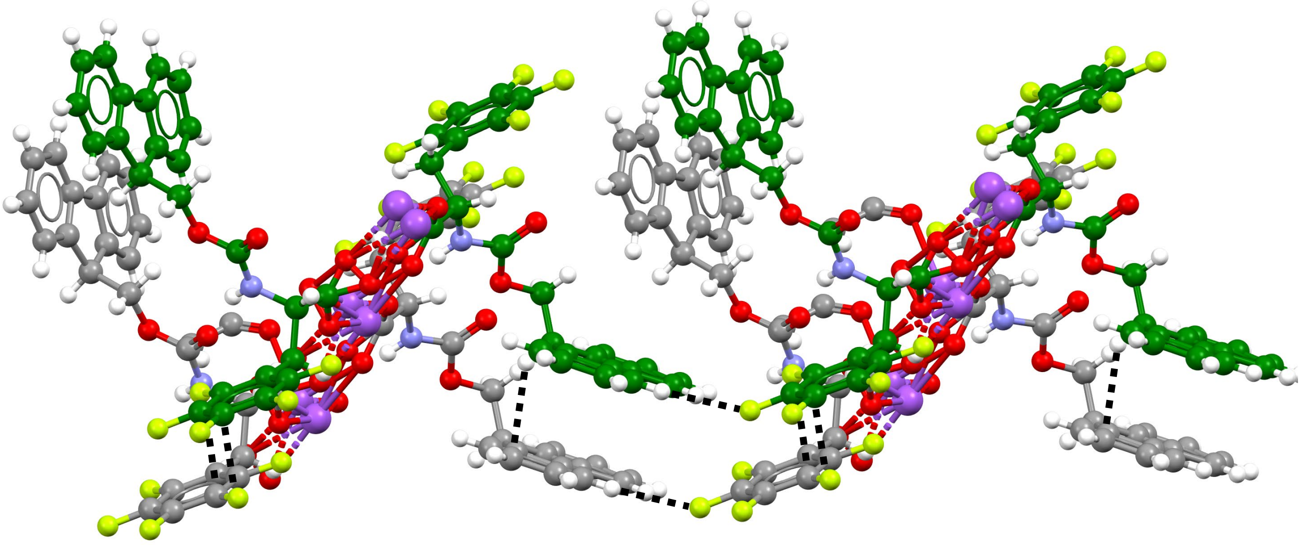

**Figure S9.** Ball-and-stick partial view of the crystal packing of the inter-chain packing in **$F_5$·Na**. Stacked piles of amino acid residues interacted laterally through a network of C-H···F and C-H···π contacts involving Fmoc moieties and perfluorinated rings, indicated by black dotted lines. Color code: carbon, gray; oxygen, red; nitrogen, light blue; fluorine, yellow; sodium and potassium, violet.

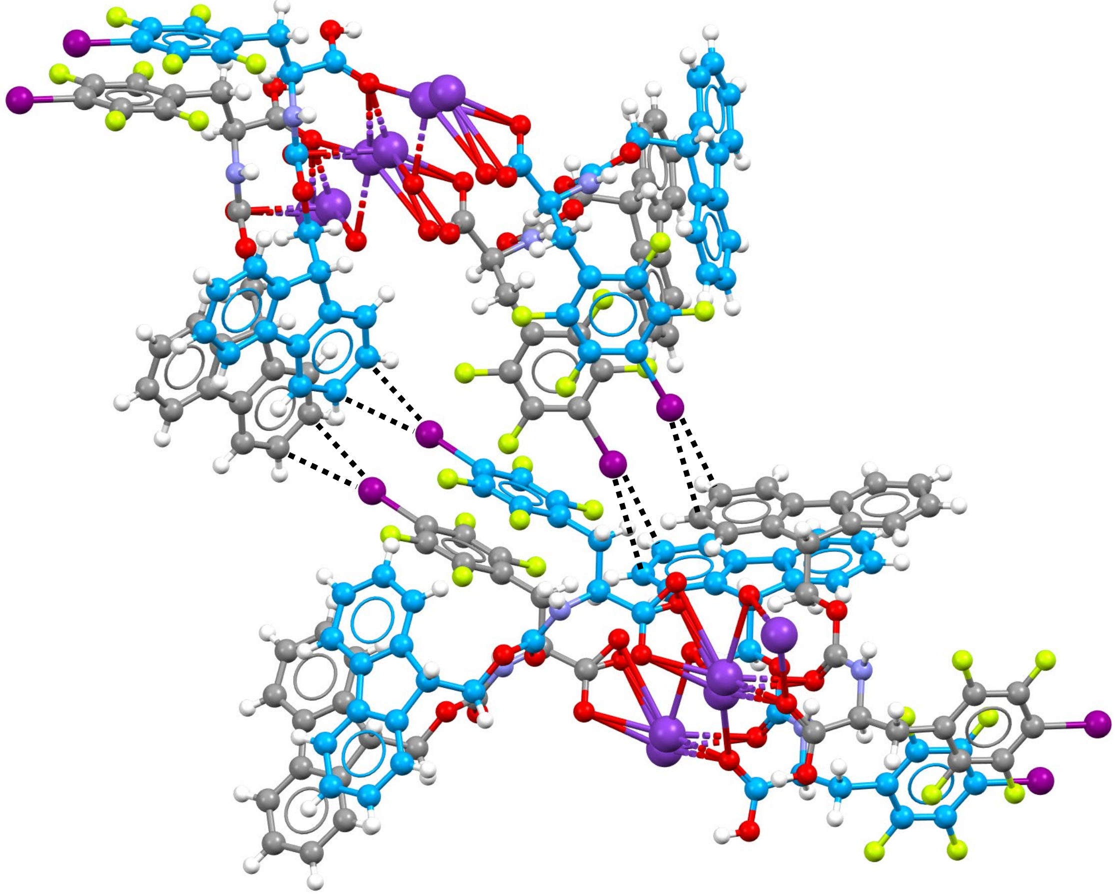

**Figure S10.** Ball-and-stick partial view of the inter-chain crystal packing of **$IF_4$·K**. XB I···π interaction between iodine atoms and Fmoc groups are shown as black dotted lines. Color code: carbon, gray; oxygen, red; nitrogen, light blue; fluorine, yellow; iodine, purple; sodium and potassium, violet.

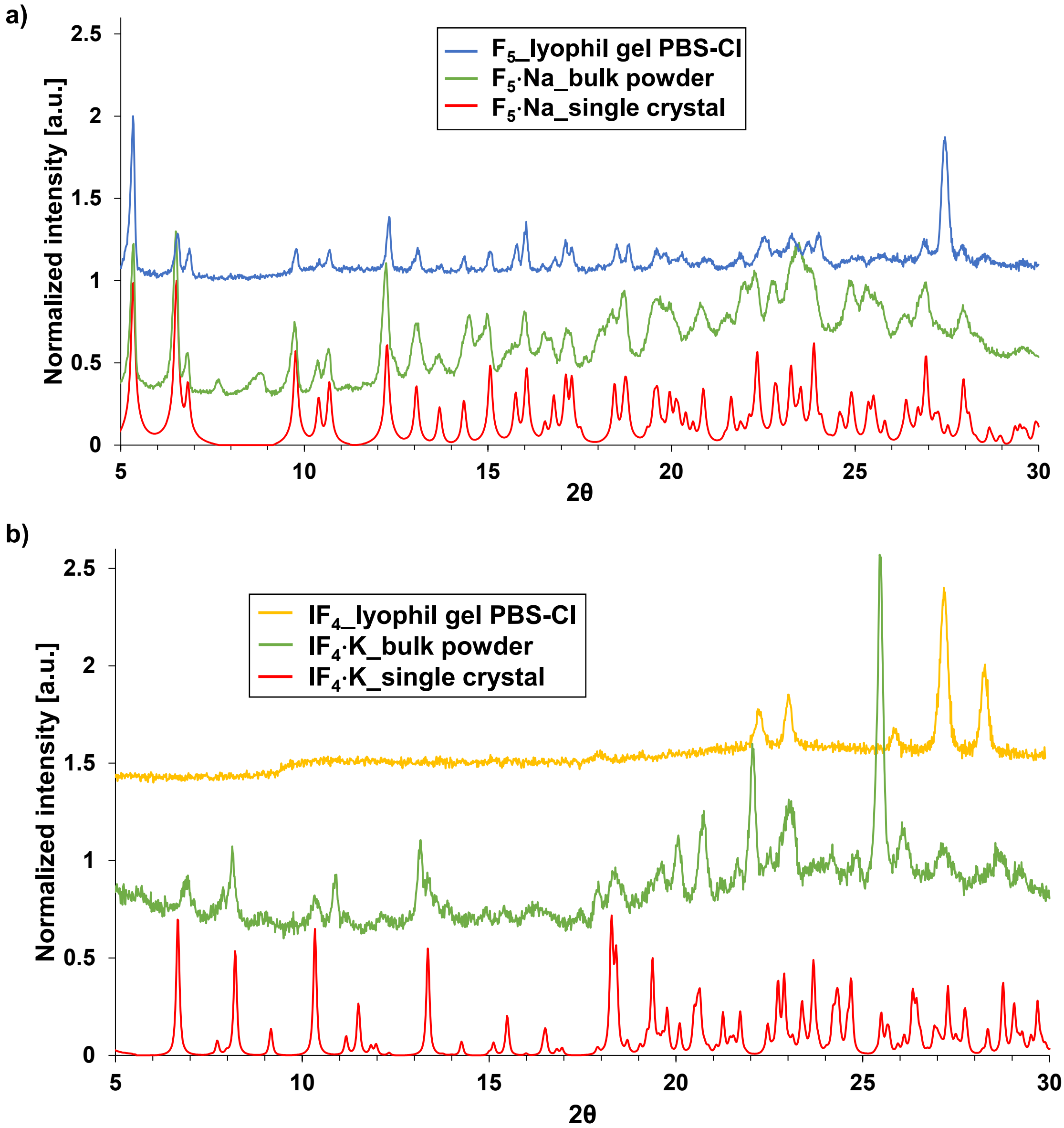


**Figure S11.** Comparisons among PXRD patterns of crystals of amino acids' alkali metal salts (simulated from single crystal), their bulk milled powders (experimental) and lyophilized hydrogels from PBS-Cl of pristine amino acids (experimental) of: a) $\mathbf{F_5}$; b) $\mathbf{IF_4}$.

## 7. References

S1. G. Bergamaschi, L. Lascialfari, A. Pizzi, M. I. Martinez Espinoza, N. Demitri, A. Milani, A. Gori, P. Metrangolo, *Chem. Commun.* **2018**, *54*, 10718-10721.
S2. A. Lausi, M. Polentarutti, S. Onesti, J. R. Plaisier, E. Busetto, G. Bais, L. Barba, A. Cassetta, G. Campi, D. Lamba, A. Pifferi, S. C. Mande, D. D. Sarma, S. M. Sharma, G. Paolucci, *Eur. Phys. J. Plus* **2015**, *130*, 43, 1-8.
S3. W. Kabsch, *Acta Crystallogr. D* **2010**, *66*, 125-132.
S4. J. Agirre, M. Atanasova, H. Bagdonas, C. B. Ballard, A. Basle, J. Beilsten-Edmands, R. J. Borges, D. G. Brown, J. J. Burgos-Marmol, J. M. Berrisford, P. S. Bond, I. Caballero, L. Catapano, G. Chojnowski, A. G. Cook, K. D. Cowtan, T. I. Croll, J. E. Debreczeni, N. E. Devenish, E. J. Dodson, T. R. Drevon, P. Emsley, G. Evans, P. R. Evans, M. Fando, J. Foadi, L. Fuentes-Montero, E. F. Garman, M. Gerstel, R. J. Gildea, K. Hatti, M. L. Hekkelman, P. Heuser, S. W. Hoh, M. A. Hough, H. T. Jenkins, E. Jimenez, R. P. Joosten, R. M. Keegan, N. Keep, E. B. Krissinel, P. Kolenko, O. Kovalevskiy, V. S. Lamzin, D. M. Lawson, A. A. Lebedev, A. G. W. Leslie, B. Lohkamp, F. Long, M. Maly, A. J. McCoy, S. J. McNicholas, A. Medina, C. Millan, J. W. Murray, G. N. Murshudov, R. A. Nicholls, M. E. M. Noble, R. Oeffner, N. S. Pannu, J. M. Parkhurst, N. Pearce, J. Pereira, A. Perrakis, H. R. Powell, R. J. Read, D. J. Rigden, W. Rochira, M. Sammito, F. Sanchez Rodriguez, G. M. Sheldrick, K. L. Shelley, F. Simkovic, A. J. Simpkin, P. Skubak, E. Sobolev, R. A. Steiner, K. Stevenson, I. Tews, J. M. H. Thomas, A. Thorn, J. T. Valls, V. Uski, I. Uson, A. Vagin, S. Velankar, M. Vollmar, H. Walden, D. Waterman, K. S. Wilson, M. D. Winn, G. Winter, M. Wojdyr, K. Yamashita, *Acta Crystallogr. D* **2023**, *79*, 449-461.
S5. P. R. Evans, G. N. Murshudov, *Acta Crystallogr. D* **2013**, *69*, 1204-1214.
S6. G. M. Sheldrick, *Acta Crystallogr. A* **2015**, *71*, 3-8.
S7. G. M. Sheldrick, *Acta Crystallogr. C* **2015**, *71*, 3-8.
S8. P. Emsley, B. Lohkamp, W. G. Scott, K. Cowtand, *Acta Crystallogr. D* **2010**, *66*, 486-501.
S9. L. Farrugia, *J. Applied Crystallography* **2012**, *45*, 849-854.
S10. L. L. C. Schrödinger, 2015, The PyMOL Molecular Graphics System. Schrodinger, LLC. http://www.pymol.org.
S11. C. F. Macrae, I. Sovago, S. J. Cottrell, P. T. A. Galek, P. McCabe, E. Pidcock, M. Platings, G. P. Shields, J. S. Stevens, M. Towler, P. A. Wood, *J. Applied Crystallography* **2020**, *53*, 226-235.
S12. C. C. Lee, A. Nayak, A. Sethuraman, G. Belfort, G. J. McRae, *Biophys. J.* **2007**, *92*, 3448-3458.
S13. D. M. Ryan, T. M. Doran, B. L. Nilsson, *Langmuir* **2011**, *27*, 11145-11156.
S14 A. L. Spek, *Acta Crystallographica Section C* **2015**, *71*, 9-18
